# Exploring the impact of age, and body condition score on erythrocytic B_1_-Dependent transketolase activity in cats: A comprehensive analysis of thiamine status

**DOI:** 10.1016/j.heliyon.2024.e34188

**Published:** 2024-07-09

**Authors:** Andrea J. Fascetti, Jennifer A. Larsen, Angela Min, Maya Nair, Maria Montano, Cecilia Giulivi

**Affiliations:** a Department of Molecular Biosciences, School of Veterinary Medicine, University of California Davis, Davis, CA, United States; b MIND Institute, University of California at Davis Medical Center, Sacramento, CA, United States

## Abstract

One of the key factors influencing aging and morbidity is the overall antioxidant status and regenerative capacity. In examining contributors to the antioxidant status, we analyzed the thiamine status in felines and the influence of age, gender, and body condition score. We measured erythrocytic B_1_-dependent specific transketolase (STKT) activity, an enzyme in the pentose phosphate pathway, in a group of 60 sexually intact, healthy, and specific pathogen-free felines (44 females, 16 males, aged 1–17 years) with thiamine diphosphate (TDP; 0.3 and 3 mM) and without it. Only two parameters (STKT activity with and without 0.3 mM TDP) decreased with age. After adjusting for age, statistical thresholds were established using these and other age-independent parameters, identifying 15 felines with subclinical thiamine deficiency. The red blood cell proteomics analysis revealed that the pentose phosphate shunt, glycolysis, and oxidative stress response were the most affected pathways in deficient felines, confirming the above diagnosis. Age emerged as the primary factor associated with thiamine deficiency, supported by the enrichment of neurodegenerative diseases with a proteotoxicity component; five young-adult felines showed marginal or acute B_1_ deficiency, and six were adult-mature with a more chronic deficiency, possibly linked to cognitive decline, all with an underweight to ideal body condition scores. Only three senior-adult felines were deficient and overweight-obese. Detecting thiamine deficiency emphasizes the need for more accurate reference values, the establishment of advanced preventive or therapeutic measures to enhance the well-being of aging companion animals, and potential extensions to human health, particularly concerning cognitive function.

## 1 Introduction

Over the last two decades, there has been a global increase in the life expectancy of pet cats. The American Veterinary Medical Association has reported that there are currently 58.3 million cats residing in the United States, according to the 2017–2018 pet demographics [1]. In 2016, half of the cat population was over 6 years old, with 27.3 % falling between 6 and 10 years and 18.5 % aged 11 years or older. This represents a 1.5- to 1.7-fold increase from 1987 to 2016 [2]. The morbidity of aging and related illnesses varies greatly among cats and populations due to genetics, diet, and lifestyle [3,4].

While the aging process is comparable among mammalian species, it is important to recognize unique feline differences. Elderly cats have higher energy needs as they age, unlike dogs and humans. As humans and dogs age, they gain fat and lose lean mass, but cats experience a decline in lean body mass and then a reversal around age 12 [[5], [6], [7]]. Older cats have increased energy needs and digestive challenges [6,8,9], leading to more underweight cases [10]. Cats with a high body condition score, akin to the human body mass index (BMI; [11]) adjusted by sex and age, are more prone to certain medical issues (e.g., atopic dermatitis, hypertension, diabetes, asthma, ophthalmic diseases, and allergies [12]). Understanding aging mechanisms and factors like body weight and sex is crucial for veterinary professionals and research on animal models of human aging.

Despite the unknown exact mechanism underlying aging, the age-dependent decline in mitochondrial function and increases in oxidative stress have been documented in various species [13,14]. It is noteworthy that antioxidant status diminishes with age and serves as a predictive factor for mortality in older human adults [[15], [16], [17]]. Although dietary antioxidants typically encompass vitamins E, A, and C, there is a minimal emphasis on the significance of vitamin B_1_ or thiamine.

Thiamine pyrophosphate, the active form of B_1_ or thiamine, functions as a coenzyme for various enzymes that play critical roles in energy metabolism. These enzymes include pyruvate dehydrogenase, alpha-ketoglutarate dehydrogenase, and branched-chain ketoacid dehydrogenase complexes [18]. It is also an essential cofactor of transketolase, an enzyme within the pentose phosphate shunt [18]. This pathway generates NADPH to sustain the activity of several antioxidant enzymes (i.e., thioredoxin, glutaredoxin, and glutathione peroxidase-reductase systems) and for biosynthetic processes (e.g., fatty and nucleic acids). Deficiency of intracellular thiamine results in an impairment in the aerobic oxidative synthesis of ATP [19,20] and sometimes with increases in glycolysis [21,22]. Experimental thiamine deficiency serves as a classical oxidative stress model, resulting in functional and morphological cellular damage [[23], [24], [25], [26]]. Notably, the phenotype of thiamine deficiency overlaps with various mitochondrial diseases [27,28]. Highly metabolically active organs such as the brain [29] are notably prone to thiamine deficiency due to elevated oxidative stress levels [13], reliance on mitochondria, and glucose metabolism [18].

Cats, similar to other small hypercarnivores, require gluconeogenic amino acids for glucose-dependent tissues like the brain [30], affecting their thiamine needs. Thiamine, in its unphosphorylated form, influences membrane ion channels, nerve conduction, and synaptic transmission [[31], [32], [33]]. Detecting early feline B_1_ deficiency poses a challenge due to vague symptoms like anorexia and vomiting [34]. Some cats may experience irregular cardiac patterns [35]. Without treatment, it can progress to neurological issues such as ventroflexion, blindness, ataxia, vestibular signs, altered behavior, seizures, coma, and death [[36], [37], [38], [39]].

Most studies documenting feline B_1_ deficiency are diet-related, such as foods containing substances that inactivate thiamine (e.g., thiaminase and sulfites, heat) or with insufficient thiamine content [34,37,38,[40], [41], [42], [43], [44], [45], [46], [47], [48]]. A presumptive diagnosis of B_1_ deficiency is usually made based on medical and diet history (including dietary thiamine concentrations), clinical and neurological presentation, and imaging findings. Sometimes the rapid clinical response to B_1_ supplementation [38,46,49,50]. Various methods of assessment of thiamine status are utilized in humans and laboratory animals, including direct measurement of thiamine in blood and urine and functional evaluation [e.g., erythrocyte transketolase (TKT) activity]. While direct approaches have been advocated, progress in veterinary research and clinical use has been hindered by methodological challenges (e.g., TKT enzymatic analysis), lack of reference laboratory testing availability (both approaches), and reliable reference ranges (both approaches). Therefore, currently, absolute confirmation relies on the demonstration of reduced TKT activity in red blood cells or other indirect methods, including urinary organic acid analysis and dietary analysis [19,[51], [52], [53]]. However, thiamine concentrations in biological fluids do not reflect its biological impact as an enzyme cofactor of TKT. Thus, thiamine concentrations are not a metabolic functional endpoint, and its blood concentration does not determine the saturation of TKT for developing full activity. Moreover, blood thiamine content does not correlate with TKT activity at initial steps (or subtle) thiamine deficiency [54]. On the other hand, TKT activity is a functional measure of thiamine status.

To bridge this knowledge gap and better understand thiamine status in cats, we evaluated a series of B_1_-related parameters or ratios for each blood sample. Among them are the specific TKT activity (STKT), primary activation ratio (PAR), latency, further activation ratio (FAR), selective activation ratio (SAR), and selective activation difference (SAD).

**Fig. 1:**
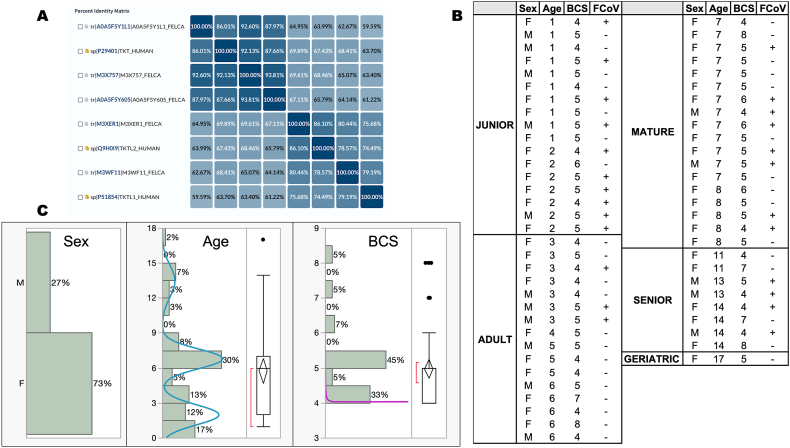
*Transketolase isoforms and demographics of the study cohor*t A. Protein sequences for human or cat TKT or isoforms were obtained from UniProt database (release 2024_02). Three reviewed proteins from humans were obtained (TKT, TKTL1, and TKTL2), and five unreviewed entries were obtained under “transketolase” filtered by *Felis catus* (under taxonomy), all inferred from cat genome studies and homology [69,70]. They were aligned using the Clustal Omega Program. Three feline proteins show high homology to each other (88–94 %) and to the human TKT (86–92 %; Fig. 1A). The other two feline proteins have an 80 % homology, lower homology to TKT (62–69 %), but high homology to their human TKTL1 and TKTL2 counterparts (79 % and 86.1 %, respectively). The matrix shows the percent identity (numbers), and the intensity of the shade is indicative of the percentage (the higher, the darker). The identification of each protein was extracted from UniProt indicating database (unreviewed in TrEMBL or tr; or reviewed in UniProtKB-SwissProt), accession number, and species. B. The cats in the study cohort were divided into 5 categories according to age (junior, adult, mature, senior, and geriatric). Each age category shows the sex (F or M for female and male, respectively), age (in years), body condition score (BCS), and feline coronavirus test (+ and -, positive and negative). C. Distribution of the sex, age, and BCS of the study cohort. Age and BCS data did not follow a normal distribution but a normal 3 mixture (age) and a SHASH one, respectively (BCS; **Supplementary Information**).

The gold standard for detecting thiamine deficiency is activating transketolase activity (TKT) with TDP supplementation. This method, known as the thiamine diphosphate effect and calculated as PAR [51,[55], [56], [57], [58], [59]], is better than assessing STKT because it can identify mild thiamine deficiency in at-risk patients. The reasons behind this effect could be the separation of the cofactor from the holoenzyme or the presence of TKT isoforms with lower TDP affinity. According to research, FAR, SAD, and SAR can identify TKT forms with unusually low TDP affinity [[60], [61], [62], [63], [64]], potentially indicating a more long-term B1 deficiency [65]. The human protein database has three TKT proteins—TKT, TKTL1, and TKTL2—from different genes. TKTL1 shows a greater preference for xylulose-5-phosphate as a substrate compared to TKT [66,67], which might have lower TDP affinity [68], making it more susceptible to thiamine deficiency. These discoveries, along with the presence of TKT isoforms in cats with relatively high homology to those in humans (Fig. 1A), support the use of other thiamine-related markers.

In addition, both TKT and glutathione reductase (GR) are integral to cellular defense against oxidative stress. As indicated above, TKT contributes to the regeneration of NADPH, a crucial cofactor for antioxidant systems. GR, reliant on riboflavin (vitamin B_2_), directly maintains the reduced form of glutathione, a key antioxidant molecule that scavenges reactive oxygen species. Therefore, deficiencies in either B_1_ or B_2_ compromise the cell's ability to combat oxidative stress, underscoring the interconnectedness of these vitamins in maintaining redox balance. To address this issue and discern between generalized vitamin B vs. thiamine deficiencies, we also tested the activity of GR as a marker for B_2_ status and as part of the antioxidant system.

Assessing vitamin B_1_ levels in elderly cats is crucial for dietary support and health prevention. The study examines B_1_-dependent transketolase as an indicator of B_1_ status in aging cats, aiming to establish targeted intervention guidelines. This pioneering research evaluates age-related effects on B_1_ status in a controlled cat population, accounting for variables such as gender and body condition.

## 2 Results and discussion

### 2.1 Cohort description

Data and blood samples were collected at a single time point from all cats present in the facility from April 2020 through July 2021. Ten were analyzed at two-time points (3–5 months apart) with no statistical significance on the enzymatic tests. The 60 cats were specific-pathogen-free, sexually intact (44 females, 16 males; Fig. 1B and **C**) ranging from 1 to 17 y old (mean ± SD = 6 ± 4; Fig. 1B). To put the ages in the context of humans, most cats were between 15 and 57 y old (average ∼40 y old; [71]), with the rest older than 61 y. The cohort had an almost equal representation of junior (1–2 y; n = 17), adult (3–6 y; n = 16), and mature cats (7–10 y; n = 18) with less representation of senior (11–14 y; n = 8) and geriatric cats (≥15 y; n = 1). As such, age did not follow a normal distribution, but a normal 3 mixture (Fig. 1C) with 1 outlier (cat aged 17 y).

In terms of the overall assessment, all cats included in this study underwent veterinary examinations and were determined to be “apparently healthy” (even if few tested positive for feline coronavirus; see Methods; Fig. 1B) with no obvious phenotypic signs of thiamine deficiency (e.g., anorexia, vomiting, ataxia, cervical ventroflexion).

The thiamine content of the diet (average of 200.3 mg thiamine/kg dry matter), tested before the study started, exceeded by 40-fold the current Association of American Feed Control Officials minimum for all life stages of cats (set at 5.0 mg/kg dry matter) [72]. 10.13039/100014337Furthermore, this diet supported a consistently high level of reproduction and growth in the colony for over 10 generations.

The body condition scores (BCS) were used as a semiquantitative method of evaluating body composition. Using this system, each unit decrease or increase in body condition score below or above ideal is approximately equivalent to 10–15 % under or over ideal body weight [73]. To provide context for this statement, the body condition score of cats is usually examined with a 9-point system indicating 1/9, emaciated; 2/9, very thin; 3/9, thin; 4/9, underweight; 5/9, ideal; 6/9, overweight; 7/9, heavy; 8/9, obese; 9/9, severely obese; [11,74]. The BCS relies on visual assessment and palpation of specific body regions, including the rib cage, waist and lumbar region, and base of the tail. In the cohort, most cats had a BCS of 5 (n = 26), with the rest at either 4 (n = 18) or ≥ 6 (n = 10). The BCS did not follow a normal distribution but a Sinh-Arcsinh (SHASH) one (Fig. 1C) with a few outliers, especially on the overweight side. However, the mean value of 5 indicated that most cats had an optimal body weight (5 ± 1).

### 2.2 Thiamine status assessments

**Fig. 2:**
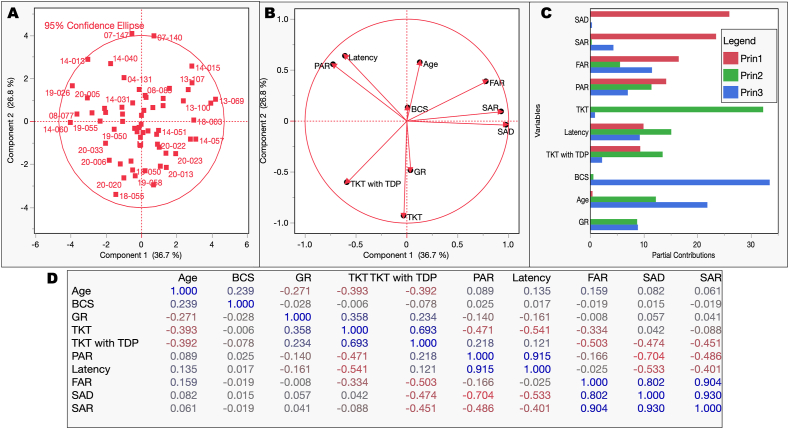
*Principal component analysis of vitamin B-related outcomes*, *age, and body condition scores*. To assess the variation in a set of variables in terms of a smaller number of independent linear combinations (principal components) of those variables, a principal component analysis (PCA) platform was applied. This statistical and machine learning procedure allowed summarizing the information in large data sets using a smaller set of “summary indices” that can be more easily visualized and analyzed. All variables were included in the analysis except sex, which was included as a supplementary one. Each variable value was centered and standardized individually, and the covariance matrix was used to obtain the principal components. The variance estimation was performed row-wise. Score (**A**) and loading plots (**B**) for the first two principal components were obtained from the principal component analysis (row-wise variance estimation) performed with all samples and data. **C**. Partial contributions of each variable to the first three principal components highlighting the weight of SAD, SAD, and FAR on principal component 1 and PAR, STKT, TKT + TDP, and latency for principal component 2. Age, BCS, and GR seemed more relevant for principal component 3. **D**. Correlation matrix among variables with blue (positive) and red (negative) being the most significant, whereas grey values are not significant.

As indicated in the Introduction, for each blood sample, GR activity and the following B_1_-related parameters or ratios were evaluated: Specific TKT activity, PAR, latency, FAR, SAR, and SAD (see details under Methods). To identify patterns in the data and represent them more meaningfully, we used principal component analysis as a statistical technique to simplify data by reducing its dimensionality. This analysis showed that most cats fell within the 95 % confidence ellipse (Fig. 2A). The outcomes were visualized in a PCA loading plot, in which the principal component 1 (PC1) had a variance of 36.7 % and that of principal component 2 (PC2) was 26.8 % (Fig. 2B). The main variables contributing to PC1 were SAD, SAR, and FAR, whereas those for PC2 were STKT, Latency, and TKT with TDP; Fig. 2C). In the loading plot, the group of 3 nearly horizontal and very tightly knit variable markers for 3 of the parameters (FAR, SAD, and SAR) indicated a group of highly correlated variables (as expected as they share the same variables), the other set was constituted by latency and PAR, and the third one by STKT, TKT + TDP and [64]. Notably, age was negatively correlated with these last 3 outcomes. Published reports indicated that STKT and PAR give comparable indications of marginal deficiency, whereas STKT was reported as the only parameter inversely correlated with age in humans [60]. The statistically relevant correlations were shown in Fig. 2D, in which the inverse correlations with age of GR, STKT, and TKT + TDP were confirmed, as well as the correlations among FAR, SAD, and SAR.

**Fig. 3:**
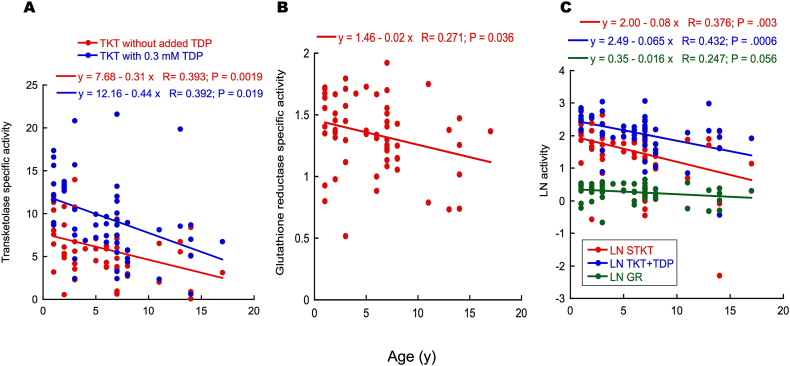
*Changes in B*_*1*_*-dependent transketolase and B*_*2*_*-dependent glutathione reductase activities with age***A.** Age-dependence decline in transketolase-specific activity. The specific activity was evaluated in feline hemolysates without (red) and with 0.3 mM thiamine diphosphate supplementation (blue). Linear regression equations are shown on top with P values from Pearson's test. **B.** Age-dependence changes in glutathione reductase-specific activity. The specific activity was evaluated in feline hemolysates under optimal conditions. **C.** Pseudo-first order kinetics of STKT, TKT + TDP, and GR activities with age. Data were LN transformed. For all panels: The linear regression equation, R^2^ (R), and P values from Pearson's test are shown at the top.

Considering that 3 variables (TKT, TKT + TDP, and GR) were correlated with age, the data were fitted to a linear regression (Fig. 3). The STKT (expressed as units/mg protein) assessed in blood samples declined steadily from 1 to 17 y of age (Fig. 3A; red; P = 00.0019). The decline in STKT activity with age was apparent in both sexes (not shown) despite the higher female-to-male ratio (F/M = 2.75). It was unrelated to hemoglobin concentrations, packed red cell volumes, or Heinz bodies’ evaluation.

The B_1_-supplemented TKT (TKT + TDP) declined with age, similarly to that observed in STKT (Fig. 3A; blue; P = 00.019). The inverse correlation of TKT activity and age was also reported for humans but presented a slower decline: about a 25 % decrease in a 60 to 70 y period [60]. The faster decline in TKT activity in cats could be explained by species-specific differences in aging progression, exemplified above by the equivalence between human and cat years. It does not result from species-specific differences in basal metabolic rates because aging in humans is associated with a gradual decline in the maintenance energy requirement (about 20 %) resulting from lower physical activity and basal metabolic rate. In contrast, the decline is evident in cats when they reach 10–12 y of age. After this age, the evidence is controversial, with some reporting from no clear age-related change [75,76] to increases after ≥10 y of age [10,77,78].

To test whether this age-dependent TKT activity decline was B_1_-specific or shared by other B-dependent enzymes, we tested the B_2_-dependent GR activity in red blood cells [79,80]. The GR-specific activity declined significantly with age, similar to STKT and TKT + TDP (Fig. 3B; P = 00.036). Using a pseudo-first-order kinetics assumption [Activity_t_ = Activity_t = 0_ * *e*^−(k^*^t)^], the natural logarithmic values of the 3 variables were plotted against age. Both TKT and TKT + TDP linear regressions were significant (Fig. 3C; P = 0.003 and 0.0006, respectively), whereas that of GR was marginally significant (P = 0.056). The half-lives (i.e., the time it takes for each variable to be reduced by half) were 8.7, 10.7, and 43.3 y for TKT, TKT + TDP, and GR, indicating that the decline in TKT activity vs. that of GR with age was biologically more relevant considering the median lifespan of intact cats (1–19–21 y; [81]) and more specific for B_1_ than B_2_.

**Fig. 4:**
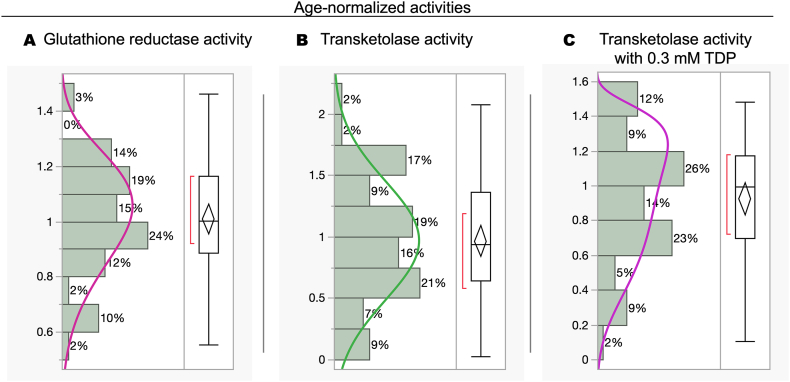
*Distribution of age-normalized glutathione reductase and transketolase activities*. The panels show the distribution of age-normalized glutathione reductase activity (A), specific TKT activity (B), and TKT with 0.3 mM TDP (C). The best-fit distribution for the data was Weibull, Normal, and SHASH, respectively (**Supplementary Information**). The data fit a distribution with a continuous variable (age). The data were fit to all continuous distributions, and by default, the best-fit distribution was selected and displayed in the histogram (shown here). In the supplementary information, the fitting list is sorted by the corrected Akaike's information criterion (AICc), information-based criteria that assess model fit, in ascending order. To establish the cut-offs that would define deficiency, for those not following normal distribution (GR and TKT + TDP), a nonparametric one-sided tolerance interval was selected, whereas for the normal one, a one-sided tolerance interval (that included 90 % of the population and setting a 1-alpha of 0.95).

According to the data presented in Fig. 3, normalizing the values by age could enhance their reliability as indicators of marginal B_1_ or B_2_ deficiencies. Both enzymatic activities were normalized by age following a previously published approach [61]. As no studies establish thiamine-deficient cut-offs for cats and to avoid an arbitrary cut-off, we identified deficient cats whose age-normalized TKT or GR in blood was below the statistical cut-off for an average population [61]. As such, the distribution of the data normalized to age was assessed to establish the best way to set a cut-off value for identifying vitamin B1 or B_2_ deficiencies. The procedure for fitting the data distribution involved utilizing the maximum likelihood algorithm, a statistical method for estimating the parameters of a probability distribution. The rationale for choosing the best fitting parameters was determined by evaluating the Akaike Information Criterion with a correction for small sample sizes (AICc) values and weights. This approach identified the most appropriate model by considering both goodness of fit and model complexity (Fig. 4; **Supplementary** Information). According to the smallest AICc, the age-normalized TKT, TKT + TDP, and GR values best fit Normal, SHASH, and Weibull distributions (Fig. 4; **Supplementary Information**; [82]). To establish the cut-offs that would define deficiency, for those not following normal distribution (GR and TKT + TDP), a nonparametric one-sided tolerance interval was selected, whereas for the normal one, a one-sided tolerance interval (that included 90 % of the population and setting a 1-alpha of 0.95). The lower tolerance interval for age-normalized GR and TKT were 0.56 and 0.08, and the upper for the age-normalized TKT with TDP was 2.38.

**Fig. 5:**
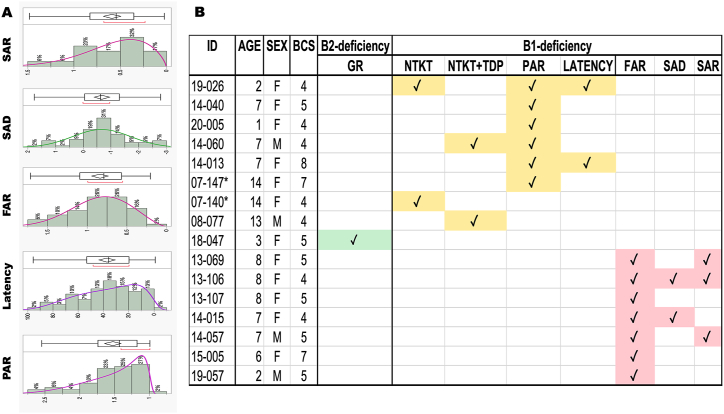
*Distribution of age-independent vitamin B*_*1*_*outcomes and identification of thiamine-deficient cats***A**. The panels show PAR, latency, FAR, SAD, and SAR distributions. PAR and Latency data followed a SHASH distribution, FAR and SAR a Weibull, whereas SAD values were normally distributed (**Supplementary Information**). As such, the one-sided tolerance intervals were calculated using nonparametric one-sided tolerance intervals. **B.** Samples identified as B_1_ (yellow or pink) or B_2_ (green) deficient. Asterisks indicate half-siblings.

Following the same analysis, we assessed the distribution of the rest (age-independent) of the variables (Fig. 5A). Since the decrease of TKT enzyme activity with age was evident regardless of its *in vitro* TDP supplementation, the activation ratios were age-independent, consistent with human studies [60].

PAR and Latency data followed a SHASH distribution, FAR and SAR a Weibull, whereas SAD values were normally distributed (Fig. 5A; Supplementary Information). As such, the one-sided tolerance intervals were calculated using a non-parametric approach. These analyses resulted in an upper tolerance interval of 3.28, 88.26, 2.62, and 2.06 for PAR, Latency, FAR, and SAR, respectively. For SAD, the upper tolerance interval was 1.88.

Using these cut-offs, 16 samples with at least one abnormal outcome were classified as “vitamin B deficient” (Fig. 5B). Only one sample was identified as B_2_ deficient, whereas the rest (n = 15) were B_1_ deficient. Of these 15 (11 females, 4 males; aged 7 ± 4 y; BCS = 5 ± 1), 8 were outside the normal range in at least one of the outcomes performed with either with or without 0.3 mM TDP supplementation (namely, age-normalized TKT, age-normalized TKT + TDP, PAR, and latency). In contrast, the rest (n = 7) were outside the range of outcomes obtained with the highest TDP concentration (FAR, SAD, and SAR). Notably, none of the samples deficient in the TKT-derived outcomes (age-normalized TKT, age-normalized TKT + TDP, PAR, and latency) overlapped with those identified as deficient with the FAR, SAR, and SAD parameters. Only 2 cats were genetically related (indicated with an asterisk) identified as half-siblings (Fig. 5B). Among those outcomes performed with 0–0.3 mM TDP, PAR was the most common parameter with abnormal values (6 of 15 samples). From those outcomes obtained with 3 mM TDP, FAR was the most common one (7 of 15).

**Fig. 6:**
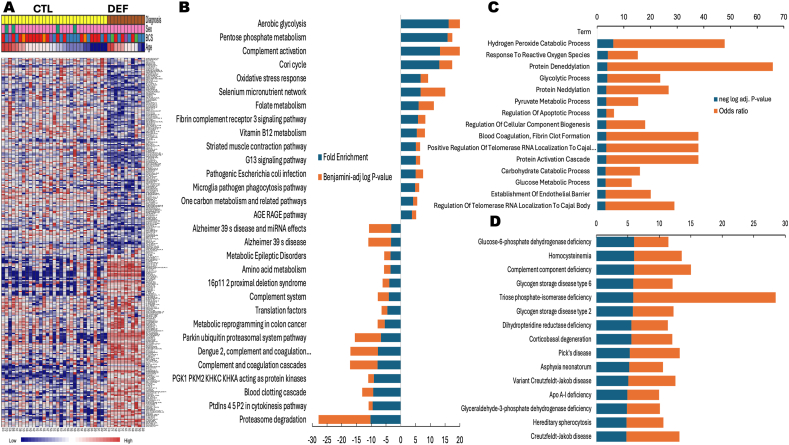
*Differentially expressed proteins, biological pathway, biological process and disease analyses in B*_*1*_*-deficient cats***A.** Untargeted proteomics was performed in 42 red blood cell samples from 31 control and 11 deficient cats from our cat cohort. DEP (n = 310; P ≤ 0.05) were represented in a heatmap with features clustered by the Ward's algorithm and samples by diagnosis or class [83]. B. Up- and down- DEP were analyzed in DAVID (https://david.ncifcrf.gov) utilizing the WikiPatwhay database. Only top 15 from each are shown (full list under **Supplementary Information**). Fold enrichment is visualized with a positive (up) or negative (down) sign, negative (up) or positive (down) log of Benjamini-adjusted P-value. **C.** Gene ontology Biological Process enrichment of DEP performed with EnrichR [84] (only top 15, full list under **Supplementary Information**). **D.** Disease enrichment performed with DEP in EnrichR and utilizing the database Rare Diseases AutoRIF Gene Lists (genes associated with rare diseases based on PubMed search). Only top 15 are shown, full list under **Supplementary Information**.

**Fig. 7:**
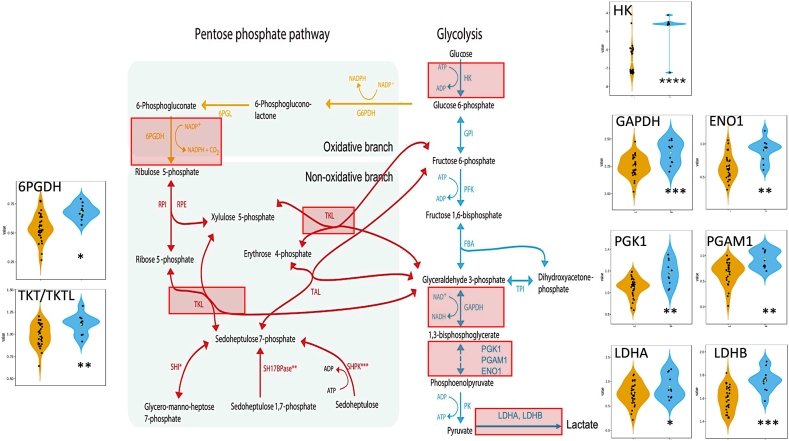
*Glycolysis and pentose phosphate shunt in deficient cats.* Based on proteomic data, schematic representation of enzymatic steps in glycolysis and pentose phosphate shunt. Red boxes, overexpressed proteins. The data distribution of DEP was depicted in violin plots for each diagnostic group using density curves (plots and statistics performed with [83]). ****P < 00.0001; ***P < 00.001; **P < 0.01; *P ≤ 0.05.

Since the animals deficient in vitamins B_1_–B_2_ were identified using statistical cutoffs of functional parameters rather than clinical signs (as none showed overt signs of thiamine deficiency), we followed an objective, independent approach to confirm their subclinical deficiency status. To this end, we conducted proteomics analysis on blood samples from those cats in our cohort with sufficient blood volume for mass spectrometry analysis (n = 42 of 60; 31 controls of 44 and 11 deficient of 16). The control or not deficient group had 31 cats, of which 27 were females (87.1 %), with 7 juniors, 10 adults, 9 mature, 4 seniors, and 1 geriatric. The “deficient group” had 11 cats, all females, 2 juniors, 1 adult, 6 mature, and 2 seniors. The (n-1) Chi-squared test indicated no significant differences between these groups regarding sex or age. Proteomics was performed in these samples, identifying 1063 proteins (**Supplementary Information**). As expected, the cell type ontology identified erythrocytes as the main cell type, followed by platelets and immune cells (**Supplementary information**). To identify differentially expressed proteins (DEP) from the 1063 identified proteins, we used the limma linear model with covariate adjustment for a multi-factor comparison analysis of complex metadata. For the differential expression analysis, we controlled for sex, age, and BCS as covariates, using diagnosis as primary metadata. The data was adjusted using robust trend, with a p-value threshold of 0.05 and no cut-off for fold change. This process resulted in 310 DEP between control (not deficient) and deficient groups, with 165 being over- and 145 under-expressed, as visualized in a heat map (Fig. 6A). To identify biological pathways related to thiamine deficiency, two pathway analysis tools were utilized (DAVID and EnrichR), providing similar results (Fig. 6B and C). As expected for putative compensatory mechanisms to overcome thiamine deficiency, over-represented pathways were glycolysis and pentose phosphate shunt (to increase NADPH output), followed by oxidative stress response and selenium micronutrient network (to account for the deficiency in NADPH-dependent antioxidant defenses). The overrepresented enzymatic steps in glycolysis and pentose phosphate shunt were indicated along with the fold change distribution of each DEP included in these pathways (Fig. 7). Under-represented pathways were constituted by proteasome and parkin-ubiquitin catabolism, suggesting accumulation of denatured, unfolded proteins (proteotoxicity) due to excess oxidative stress. Biological process enrichment supported and extended these findings with the enrichment of hydrogen peroxide catabolic process, glycolysis, and apoptosis (Fig. 6B). Diseases enriched by DEP pointed to a dysfunctional pentose phosphate shunt supported by the glucose-6-phosphate dehydrogenase, triose phosphate isomerase, and glyceraldehyde-3-phosphate dehydrogenase deficiencies (Fig. 6C). Notably, several diseases associated with proteotoxicity were obtained, likely the results of oxidative stress-mediated protein damage not accompanied by proteolytic catabolism; among them, corticobasal degeneration, Pick's disease, and Creutzfelt-Jakob diseases.

Although a subset of samples was utilized for the proteome analysis (70 %), these findings distinctly and conclusively substantiated the identification of thiamine deficiency, as inferred from the specified functional criteria.

**Fig. 8:**
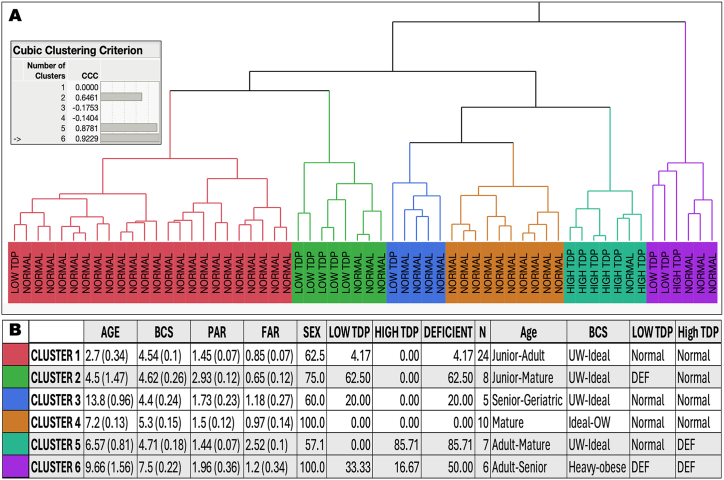
*Hierarchical clustering of selected vitamin B*_*1*_*outcomes and cluster information***A**. Hierarchical clustering (center; Ward method, robust column standardization) shows 6 clusters according to the cubic clustering criterion (identified with colors). Each cluster was analyzed regarding age, BCS, PAR, and FAR by using ANOVA followed by Fisher LSD (full analysis shown in **Supplementary Information**). The Distance Graph is the plot that appears above the dendrogram. This graph has a point for each step where two clusters are joined into a single cluster. The vertical coordinates represent the number of clusters, which decrease from left to right. The horizontal coordinate of the point is the distance between the clusters that were joined at the given step. The dendrogram is represented using a distance scale. **B**. Statistcial analyses of each cluster in terms of age, BCS, PAR, FAR, and sex. Numbers in age, BCS, PAR and FAR represent mean (SD). Sex is shown as the percentage of females. LOW or HIGH TDP were based on the statistical cut-offs, and calculated as the percentage of each cluster. DEFICIENT is the percentage of cats deficient regardless of low or high TDP. N is the number of cats/cluster. Description of Age, BCS, LOW, and HIGH TDP in words took into consideration the definitions indicated in the main text and the lower and upper 95 % confidence interval values.

As the proteomics results were obtained with a subset of samples, the entire cohort was analyzed using a hierarchical cluster, adding PAR as a surrogate for “low TDP” and FAR for “high TDP”, in addition to BCS, sex, and age, to elucidate the presence of endophenotypes. Clustering, a multivariate technique, groups observations that share similar values across several variables to understand the clumping structure of our cohort. The method begins by treating each observation as its cluster. Then, at each step, the two clusters that are closest in terms of distance are combined into a single cluster. The result is depicted as a tree, called a dendrogram. In our case, the analysis was performed using the Ward method and robust column standardization (Fig. 8A). The dendrogram depicted 6 clusters according to the cubic clustering criterion (Fig. 8A; **Supplementary Information**). Consistent with the results obtained with proteomics and functional outcomes, clusters 2 (low TDP; highest average PAR values), 5 (high TDP; highest average FAR values), and cluster 6 (second with highest PAR and FAR values) had the highest incidence of cats deficient in B_1_–B_2_ (62.5, 85.7, and 50 %, respectively). The main variable for all three clusters was age, as each cluster was constituted mainly of adult to mature cats [(average ± SEM) cluster-2 = 4.5 ± 1.5 y old; cluster-5 = 6.6 ± 0.8 y old; cluster-6 9.7 ± 1.6 y old; Fig. 7B). In addition, cluster 6 had heavy to obese, adult-senior cats, the only cluster with vitamin B deficiency influenced by BCS.

To confirm that age was one of the main factors contributing to the deficiency diagnosis, a response screening test (utilizing a Huber robust fit) was performed using the proteomics data as variables (**Supplementary Information**). The model used a full factorial effect among diagnosis, sex, age, and BCS. Using a cut-off of 2 for the FDR logworth, the results indicated that most proteins fell into the diagnosis category (n = 101), followed closely by the diagnosis*age interaction (n = 91), with an overlap of 21, indicating the relevance of age in the diagnosis (**Supplementary Information**). The diagnosis*age*BCS and diagnosis*BCS interactions each had eight proteins, supporting the notion that BCS played a secondary role in thiamine deficiency.

## 3 Concluding remarks

Aging and morbidity are influenced by antioxidant status and regenerative capacity. Here, for the first time, functional biomarkers of thiamine status in felines were studied, considering age, sex, and BCS in a controlled cat cohort (60 sexually intact cats raised under specific-pathogen-free conditions, fed an adequate thiamine diet, without overt signs of B_1_ deficiency). Erythrocytic B_1_-dependent STKT activity was assessed in 60 healthy felines, showing age-related STKT activity variation with TDP levels. Subclinical thiamine deficiency affected 15 felines, impacting pathways like pentose phosphate and glycolysis. Age was the primary factor, with young adults showing acute deficiency and older cats with chronic deficiency.

Unlike previous studies, our study analyzed B_1_ and B_2_ status using functional biomarkers in a well-defined cat colony, avoiding the limitations of presumptive diagnoses. This approach enabled the evaluation of potential correlations between circulating or tissue B_1_-dependent activity rather than relying on indirect methods such as gene expression of B_1_-linked enzymes or direct measurements of circulating vitamin levels in biological fluids. These considerations are particularly important due to the variable bioavailability of different vitamins, the potential for impaired digestion or food intake in older humans and cats [9,[85], [86], [87]], and the risks associated with neutering, such as obesity and related health issues [88,89].

When evaluating the B_1_ and B_2_ status, our research underscores the significance of considering age to establish precise reference ranges and nutritional requirements for assessing TKT activity with and without TDP supplementation and GR activity as functional biomarkers. Using functional biomarkers adjusted for age, our study successfully identified subclinical thiamine-deficient subjects by applying statistical thresholds, validating the effectiveness of our methodology. Independent confirmation of subclinical vitamin B_1_ deficiency was obtained through a proteomics investigation, highlighting glucose metabolism and the pentose phosphate pathway as primary contributors in deficient samples. Within our investigation, PAR and FAR emerged as the predominant indicators of marginal and chronic thiamine deficiency, respectively, among adult to mature cats exhibiting optimal BCS (clusters 2 and 5). Notably, in the human context, both the specific TKT activity and PAR exhibit similarities, with only the former showing an inverse correlation with age. Cluster 6 comprises mature, heavy to obese cats displaying deficiencies in PAR and FAR, indicating an interaction between age and BCS.

Although the mechanisms underlying the decline in TKT activity with age were not explored in this or any other study, there are some scenarios that could be excluded. The decline in TKT activity cannot be linked to an age-dependent drop in food (and B_1_) intake. While food intake was not measured in our cohort, and its contribution cannot be evaluated, variations in energy requirements resulting in thiamine deficiencies have not been recognized in cats. They are not expected to significantly contribute to thiamine status in the population evaluated in the present study, as most cats had optimal BCSs. Thus, it is improbable that the cause of B_1_ deficiency is age-related deficits in digestion, absorption, and metabolism as cats in cluster 6 were heavy to obese.

It is possible that as animals age, there is an overall decline in glucose utilization in the red blood cells, including the pentose phosphate pathway. This process may be compounded in those cats that have high BCS (cluster 6), as glucose utilization is impaired in overweight/obese subjects [90]. In support of this hypothesis, reduced activities of hexokinase and glucose-6-phosphate dehydrogenase with aging were observed in mice [91], humans [92,93], and rabbits [94]. The decline in these enzymes likely contributes to lower glycolytic (lower ATP production) and pentose phosphate pathway (NADPH) utilization rates in aged red blood cells [91]. Alternatively, age-dependent increases in oxidative stress may promote direct (e.g., oxidation of methionine residues) or indirect (e.g., covalent binding of Lys to malondialdehyde) oxidative stress modifications of chemical moieties involved in thiamine binding or homodimer dimerization, increase aggregation, proteolysis, or instability of the apoenzyme can result in decreased TKT activity [[95], [96], [97], [98], [99], [100], [101], [102], [103]], requiring higher TDP supplementation to unveil the deficiency. Finally, as indicated before, it is possible that there is a different expression of TKT isoforms with age that might explain the findings with FAR, SAR, and SAD obtained at higher levels of TDP. Although in the feline protein database, three TKT isoforms comparable to the human ones were observed (Fig. 1A), our proteomic study did not result in any peptide that cross-referenced to TKTL1 or TKTL2.

In human clinical practice, an abnormally high value of FAR (or of the derived parameters SAR or SAD) might indicate a possible cumulative effect of a chronic B_1_ deficiency and give a helpful warning of the risk of brain function decline in cats as they age. This is emphasized by the finding of neurodegenerative diseases with a proteotoxicity component (Fig. 6C). High FAR values were observed in human patients likely to be under-or malnourished, especially elderly ones, including those with dementia [65]. It is essential to realize that FAR's information was largely independent of and additional to that provided by the standard test (PAR) rather than an alternative, for no samples were abnormal in both parameters. This means that FAR can be expected to give a more broadly based warning of susceptibility to brain dysfunction or decline with age and should have a place in any clinical setting intended to prevent brain function decline occurring among elderly demented or other patients, especially at risk from thiamin deficiency.

Notably, none of the cats identified as B_1_ or B_2_ deficient overlap those reported as B_6_ deficient in our previous study [104], indicating the low likelihood of a generalized vitamin B deficiency.

An interesting application of the age-dependent decline of erythrocytic TKT activity would be to use it as an alternative to current age-estimation methods [[105], [106], [107], [108]] supported by human studies [60]. However, we need to point out that other factors may contribute to differences between the biological vs. chronological age of the cats (e.g., nutrition, stress) and that our cohort was maintained under optimal conditions, which may not reflect the cat population at large.

An altered B_1_ status may indicate conditions beyond variable energy needs and body weight, such as subclinical diabetes with or without neuropathy, hepatic dysfunction, or deficiencies in thiamine phosphorylation. The cats underwent normal physical examinations throughout the study and other daily observations remained unremarkable before, during, and after the sampling period. Therefore, this straightforward test, accurately reflecting the thiamine nutrition status at the time of blood collection, can serve as an early indicator of subclinical pathologies that require veterinary attention. Detecting thiamine deficiency through this test often poses challenges in establishing a direct causal link between the deficiency state and the clinical signs and symptoms. Nonetheless, if administering B_1_ alone leads to increased TKT activity, reduced PAR and/or FAR effect, and prompt clinical recovery [38,45,109], it is reasonable to infer that thiamine deficiency played a role in the illness.

Assessing thiamine status in aging cats is essential due to the crucial role of this vitamin in medicine and nutrition. The swift resolution of symptoms following B_1_ supplementation indicates its importance [38,45,109]. Dietary thiamine in substantial amounts, compared to pharmacological doses, can prevent adverse health effects. Thiamine-rich diets or supplementation have been reported to obscure the diagnosis or improve the phenotype in patients with *Slc19a2* deficiency [110], thiamine-responsive maple syrup urine disease [111,112], and biotin-thiamine-responsive basal ganglia disease [113]. The lack of phenotype may be attributed to the significant thiamine reserve in the brain [114,115] and the requirement for thiamine levels to be less than 20 % of normal before overt encephalopathy [114].

## 4 Limitations of the study

First, the data were collected from a single feline facility and included associated animals housed together, potentially introducing a selection bias. The lack of a sex impact may be explained by a significant predominance of female subjects in the group. However, utilizing a single data source also functions as a strength in this study, offering thorough control over numerous factors such as diet, living conditions, and specific pathogen-free status. Additionally, the accurate diagnoses of medical conditions and body condition evaluations relied on the clinical proficiency of colony veterinarians. The limited number of cats with inadequate B_1_ levels imposed constraints on certain analyses, in combination with ethical and technical limitations in repeated blood sample collection from the same individuals. The semi-quantitative assessment of BCS, categorizing superficial physical condition into specific ordinal values [116], may lead to data loss by assuming equal health risks for conditions with the same levels of excess fat. Furthermore, unlike the human BMI, setting thresholds for underweight, optimal weight, overweight, and obesity [11] in feline BCS lacks substantial epidemiological validation concerning their health implications [12]. The absence of records on food intake could have influenced B_1_ deficiency; however, most cats maintained satisfactory body condition scores, indicating adequate nutrition. The lack of comprehensive monitoring or retrieval of food samples during the observation period was addressed by pre-study assessments of thiamine content, ensuring sufficient levels of thiamine. Recognizing animals as 'apparently healthy' based on physical examinations has limitations; even with thorough screening, the complete absence of disease cannot be guaranteed by any practitioner.

Future studies should explore whether different age groups of domestic cats have varying dietary thiamine needs. More extensive research involving a larger cat population is necessary to validate and broaden the findings. Aging cats are more prone to illnesses, affecting their longevity and well-being. It is crucial for both cat owners and veterinarians to be aware of the dangers of feeding older cats an imbalanced diet, particularly raw fish and commercial foods not formulated for complete nutrition.

## Ethics declaration

The University of California, Davis Institutional Review Board for the Protection of Animal Welfare (#21780) approved all experimental procedures and the ethics of this study. The study was carried out in compliance with the IUCAC requirements and the ARRIVE guidelines [117].

All listed authors made substantial contributions to the following: (1) the conception and design of the study, the acquisition of data, or the analysis and interpretation of data; (2) drafting the article or critically revising its important intellectual content; (3) final approval of the version submitted. The contributions of individual authors were indicated in the text.

Artificial intelligence technology was used to improve readability and language (Grammarly).

## Funding disclosure

The study was supported by the 10.13039/100016512Center for Companion Animal Health, 10.13039/100009751School of Veterinary Medicine, University of California, Davis (to 10.13039/100014710CG), and discretionary funds from C.G.

## Animal subjects

The cohort used in this study has been described in detail in our previous study [104]. The experimental protocol followed the Guide for the Care and Use of Laboratory Animals (NRC 1985) and was approved by the Animal Use and Care Administrative Advisory Committee of the University of California-Davis. The study included a population of 60 sexually intact, specific-pathogen-free domestic shorthair cats (Felis catus) aged 1–17 years from the Feline Nutrition and Pet Care Center School of Veterinary Medicine, the University of California-Davis. Pathogens tested in this group included feline calicivirus, feline herpes virus, feline leukemia, and feline immunodeficiency virus (all negative). All animals in the colony between March 2020 and July 2021 were part of this study, with these criteria established beforehand. All cats underwent a veterinary examination and were found to be in apparent good health and capable of participating in the study.

Cats in this facility—as detailed before [104]—are housed in large group enclosures with enrichment. Cats were group-housed in large wire cages (2.5 × 2.5 × 2.5 m) with enrichment and in humidity- and temperature-controlled rooms (21 ± 2 °C) with a light: dark cycle of 14 h:10 h. The cats had habitual free access to tap water and a commercially available, balanced, dry expanded diet formulated for all life stages (Purina Cat Chow Complete Formula, Nestle Purina PetCare Company, St. Louis, MO). The diet samples were analyzed in duplicate for thiamine by a reference laboratory (Eurofins Scientific, Des Moines, IA) for moisture content via AOAC International Official Method 930.15 and thiamine concentration via AOAC International Official Method 942.23 [118].

This study defined “apparently healthy cat” in the context of a physical exam for all cats included in the analysis. A comprehensive assessment of the factors listed below helped veterinarians determine the overall health status of the cats, a widespread practice in the veterinary medicine literature (e.g., Refs. [[119], [120], [121], [122], [123], [124]]). As such, at the time of blood sampling of cats within the cat colony, none had a remarkable medical history, no known illness, and had normal physical examination findings with no evidence of systemic disease requiring treatment, e.g., arthritis, diabetes, thyroid disorder, liver, or renal impairment. An apparently healthy cat exhibited normal physiological parameters, including but not limited to (i) Vital Signs: Normal heart rate, respiratory rate, and body temperature within cat-specific ranges; (ii) Body Condition: An appropriate body weight and muscle mass for a domestic shorthair cat, age, and sex; (iii) Coat and Skin: Clean, shiny coat without excessive shedding, dandruff, or signs of parasites. Healthy skin with no redness, inflammation, lumps, or lesions; (iv) Eyes, Ears, Nose, and Mouth: Clear, bright eyes with no discharge or signs of irritation; Clean and odor-free ears, normal nasal discharge, and moist nose; Healthy teeth and gums, with no signs of dental disease or oral abnormalities; (v) Mobility and Posture: Normal gait, posture, and coordination; Ability to move joints and limbs without signs of pain or discomfort; (vi) Digestive System: Regular bowel movements with formed stools; Absence of vomiting, diarrhea, or signs of gastrointestinal distress; (vii) Urinary System: Normal frequency and volume of urination; Absence of blood or abnormalities in urine; (viii) Behavior and Mental State: Alert and responsive with appropriate behavior for the species; Normal social interactions and mental alertness; (ix) Reproductive Health: Normal reproductive organs and, if applicable, regular reproductive behaviors; (x) Vaccination and Preventive Care: Up-to-date vaccinations and preventive care measures appropriate for the cat's species and lifestyle.

The standing veterinarian evaluated the body condition score. The scale used scale ranged from 1 (emaciated) to 9 (grossly obese), with a score of 5 being ideal [11].

## Sample collection

Blood collection among subjects was conducted randomly, with the daily number of subjects bled varying according to the technician's schedule and workload, as previously mentioned [104]. The process of blood collection was efficiently carried out for most cats while they were conscious and appropriately restrained to prevent any movements that could result in damage to blood vessels or organs, leading to severe complications. Only trained personnel conducted this procedure, adhering to specific criteria to determine the safe maximum amount of blood to be drawn. For a 5 kg cat, the approximate volume of a single blood draw was less than 5 ml, in compliance with the University's IACUC guidelines. This volume is an estimation based on the cat's size, health condition, and hydration level. Following the blood collection, all cats were not returned to their enclosure until hemostasis was fully achieved, ensuring no further blood loss from the collection site. Hemostasis confirmation involved the use of gauze and direct pressure, with occasional application of pressure for several minutes post jugular vein puncture. It is advisable to collect blood from the medial saphenous, cephalic, and jugular, with a preference for the latter due to the procedure's anesthesia-free nature and the considerable experience of the trained staff in this specific technique.

Blood samples were collected using a disposable 20‐ml syringe and 20‐gauge needle into 2‐ml tubes containing K_2_EDTA (Sarstedt, Nümbrecht, Germany). For measurement of enzymatic activities and protein concentration, 6 ml of blood was aliquoted into 3 EDTA tubes (2 mL/tube). Blood samples were stored at 4 °C until analysis and were analyzed 7–8 h after phlebotomy. Blood samples were collected in the morning to avoid any putative diurnal cycles. After centrifugation at 3000×*g* at 4 °C, plasma was removed, and the equivalent 1 ml of packed red blood cells was washed in phosphate-buffered saline three times and suspended in 1 ml of a 1 % solution of Triton X‐100 double density, peroxide‐free detergent (Sigma, St. Louis, MO, USA). If needed, washed and permeabilized red cells were stored at −30 °C for a maximum of 3 months or at −70 °C for up to 18 months. After homogenization and incubation at 25 °C for 10‐min, the hemolysate was centrifuged at 13,000*g* for 10 min at 4 °C.

## Enzymatic activities' protocols

All reagents used for the enzymatic tests were of analytical grade or higher. The supernatants were prepared to measure the enzymatic activities by diluting 10–20 μl with 990-980 μl of 50 mM phosphate buffer (pH 7.5) containing 0.2 % bovine albumin and 2 mM K_2_EDTA. Using standard methods and reagents, the enzymatic activities were followed using kinetic spectrophotometry (340 nm) at 37 °C [125]. Transketolase activity was tested in technical triplicates as described before [19,52,53]. The following parameters and ratios were calculated for each blood sample: (i) Specific transketolase activity (TKT): This was the enzyme activity (without the addition of TDP) expressed in units per mg protein. (ii) Primary activation ratio (PAR): This was the ratio of the activity in the presence of 0.3 mM TDP to that in the absence of added TDP. (iii) Further activation ratio (FAR): This was the ratio of the activity in the presence of 3 mM TDP to that in the presence of 0.3 mM TDP. (iv) Selective activation ratio (SAR): This was the ratio of the further activation ratio to the primary activation ratio (FAR:PAR). (v) Selective activation difference (SAD): This was obtained by subtracting the primary activation ratio from the further activation ratio (FAR-PAR).

Glutathione reductase activity was tested in technical triplicates under optimal conditions as described before [126]. Transketolase or glutathione reductase specific activities were expressed as units of enzymatic activity per mg of total protein (1 U = 1 μmol x min^−1^) and calculated as (ΔAbs_340nm_ x (min x (6.22 μmol/ml))^−1^ = μmol x (min x ml)^−1^ x (mg of protein x ml^−1^) = U/mg. Protein determination was performed using the bicinchoninic acid method (Pierce™ BCA protein assay kit, catalog #23225).

## Red blood cell proteomics procedure

Red blood cell samples were subjected to acetone treatment to precipitate and concentrate the proteins and delipidate them. Four volumes of −20 °C analytical grade acetone (Sigma-Aldrich; St. Louis, MI) were added to each sample, mixed, and left overnight (6–8 h) at 4 °C. The samples were centrifuged at 16,000×*g* for 10 min at 4 °C. After the supernatant was discarded, the pellet was washed twice with −20 °C acetone and spun at 16,000×*g* for 10 min at 4°. The pellet was then dried under vacuum for 15 min on a SpeedVac. The pellets were solubilized in 100 μl of 6 M urea/50 mM ammonium bicarbonate, pH 8, and were subsequently treated with 2.5 μl of 5 mM dithiothreitol (DTT) and incubated for 30 min at 37 °C. Twenty μl of 5 mM iodoacetamide (IAA) was added, and the solution was incubated for 30 min in the dark. Afterward, 20 μl of DTT was added and incubated for 10 min at 20–22 °C to quench the IAA excess. A mass spectrometry grade mix of rLys-C and Trypsin Gold (Promega; Madison, WI) was added in a 1:25 ratio and incubated for 4 h at 37 °C. To dilute the urea concentration to >1 M, 600 μl of 50 mM ammonium bicarbonate was added and incubated overnight at 37 °C. The following day, the digests were placed in a Macro Spin Column (The Nest Group, Inc., Ipswich, MA) to desalt. Around 10–100 μg of the digests were subjected to mass spectrometry analysis following the protocol described by Ref. [127]. *Liquid Chromatography and Tandem Mass Spectroscopy* **-** The protein digests were randomized and analyzed using a Thermo Scientific Q Exactive Orbirtrap mass spectrometer fitted with a Thermo Scientific Proxeon Easy-nLC II HPLC and a Proxeon nanospray source at the Proteomics Facility at the University of California, Davis. The digests were loaded on a 100 μm × 25 mm Magic C18 200 Å 5U reverse phase trap where they were desalted online before being separated using a 75 μm × 150 mm Magic C18 200 Å 3U reverse phase column. The peptides were eluted using a 90-min gradient with a 300 nl/min flow rate. Am MS survey scan was obtained for the 300–1600 *m/z* range, MS/MS spectra were acquired where the top 15 ions in the MS spectra were subjected to high energy collisional dissociation, an isolation mass window of 2.0 *m/z* was used for precursor ion selection, and a 27 % normalized collision energy for fragmentation with a 5-s duration for dynamic exclusion [127]. *Protein Identification* **-** Using X! Tandem (THE GPM, thegpm.org; version X! Tandem Alanine (2017.2.1.4)) database searching all MS/MS samples to search the Felis catus database assuming digestion enzyme: trypsin, and searched with a fragment ion mass tolerance of 20 ppm and a parent ion tolerance of 20PPM, carbamidomethyl of cysteine, selenocysteine, Glu- > pyro-Glu of the N-terminus, ammonia-loss of the N-terminus, Gln- > pyro-Glu of the N-terminus, deamidated of Asn and Gln, oxidation of Met and Trp and deoxidation of Met and Trp were all specified in X! Tandem as variable modifications. Weighted spectral counting was used to determine protein abundance [127]. The criteria for protein identification-Scaffold (version Scaffold_4.11.1, Proteome Software Inc., Portland, OR) to validate MS/MS based peptide and protein identification were accepted if they could be established at greater than 88.0 % probability to achieve a less than 0.5 % FDR according to the Scaffold Local FDR algorithm. Acceptable protein identification was established at a probability greater than 5.0 % to achieve an FDR less than 5.0 %, and it contained at least 2 identifiable peptides, the probabilities of which were assigned using the Protein Prophet algorithm. Proteins with similar peptides not discernible with MS/MS were grouped to satisfy parsimony principles. *Red blood cell protein profiling-* Features with >50 % missing values were removed. Missing values were imputed using the multivariate robust principal components, which replace missing values using a low-rank matrix factorization (singular value decomposition) robust to outliers (JMP Pro v17.0). The proteomes were filtered by unannotated features in the human protein database. The decision to use the human protein database over the domestic cat proteomic database was based on data availability, annotation quality, and relevance to the research objectives. The human protein database, such as the Human Protein Atlas or UniProt, is extensively studied and well-annotated, providing detailed information on protein sequences, structures, functions, interactions, and expressions. This makes it a valuable resource for in-depth proteomic studies. In contrast, the domestic cat proteomic database has less comprehensive data and fewer annotations due to less research on cat proteomics. 10.13039/100014337Furthermore, human proteins have well-studied connections to other species, making the human protein database useful for evolutionary and comparative studies, and it is integrated with various bioinformatics tools, supporting a broad range of research queries (e.g., diseases), unlike cat-specific proteomic databases. The remaining 983 were normalized with the variance stabilizing normalization, and log 2 transformed [83]. To perform a multi-factor comparison analysis for complex metadata, we used the linear model with covariate adjustment of limma for its high-performance implementation. For the differential expression analysis, the primary metadata was the diagnosis, the reference group was control or not deficient, and all contrast (ANOVA-style) was evaluated, controlling for sex, age, and BCS as co-variates. Finally, the data was adjusted using robust trend [83] using *p*-value ≤0.05 with no cut-off fold change. The raw data, normalized data, and DEP were included in the **Supplementary Information**.

***Statistical analyses-***Enzymatic activities were assessed in technical triplicates. For all analyses, *P* < 00.05 was considered significant unless indicated. All data were analyzed using JMP Pro software (version 17.0.0). All other statistical analyses were indicated under each Figure or in the main text.

## Data availability statement

The data and datasets for this study are available in both the main text and the supplementary information. The study did not generate new unique reagents. All data generated or analyzed during this study are included in this published article and under Supplementary Information.

## CRediT authorship contribution statement

**Andrea J. Fascetti:** Writing – review & editing, Validation, Supervision, Resources. **Jennifer A. Larsen:** Writing – review & editing, Validation, Supervision, Resources. **Angela Min:** Writing – review & editing. **Maya Nair:** Writing – review & editing. **Maria Montano:** Supervision, Resources, Methodology, Investigation. **Cecilia Giulivi:** Writing – original draft, Validation, Supervision, Software, Project administration, Methodology, Investigation, Funding acquisition, Formal analysis, Data curation, Conceptualization.

## Declaration of competing interest

All authors have disclosed any financial or other interests related to the submitted work that could impact the author's objectivity or influence the article's content. These disclosures were made through a designated questionnaire during the submission process. In the Declaration of Interests, it is stated that the funder, the 10.13039/100016512Center for Companion Animal Health at the 10.13039/100009751School of Veterinary Medicine, University of California, Davis, played no role in the design, data collection, analysis, manuscript writing, or decision to publish the study's results. No financial or non-financial competing interests that could compromise this publication's objectivity, integrity, or value by influencing the authors' judgment and actions in data presentation, analysis, and interpretation were identified. Furthermore, Jennifer A. Larsen (JAL) is engaged in clinical trials sponsored by 10.13039/501100003551Royal Canin and Nestlé Purina PetCare, contributing to educational materials for Brief Media, Mark Morris Institute, and Healthy Pet magazine. She also participates as a speaker or attendee in continuing education events organized by Royal Canin, Nestlé Purina PetCare, Nature's Variety, and Hill's Pet Nutrition. Andrea J. Fascetti (AJF) is the Scientific Director of the Feline Nutrition and PetCare Center and Amino Acid Laboratory at the 10.13039/100007707University of California, Davis, providing fee-for-service research support. This involvement did not create conflicts of interest or influence the data collection or interpretation. AJF has provided advice to Synergy Food Ingredients and Clorox, and has received grants from Nutro, as well as remuneration for lectures or advisory roles with Nestlé Purina PetCare, 10.13039/501100009409Mars Petcare, and the Pet Food and Mark Morris Institutes. A nutrition resident received funding from 10.13039/100015312Hill's Pet Nutrition Resident Clinical Study Grants program, and AJF and JAL collaborated on a research project. The Veterinary Medical Teaching Hospital at UCD receives partial support for a Nutrition Technician from Nestlé Purina PetCare and veterinary nutrition program funding from Nestlé Purina, 10.13039/501100009409Mars Petcare, and 10.13039/100015312Hill's Pet Care. Additionally, Cecilia Giulivi serves as an Editorial Board Member of Scientific Reports. She has received compensation as a Field Chief Editor for Frontiers in Molecular Biosciences and honoraria for participating in NIH peer review meetings.
