# Supplementary material for: Exploring the impact of age, and body condition score on erythrocytic B1-Dependent transketolase activity in cats: A comprehensive analysis of thiamine status
Source: Heliyon. 2024 Jul 9;10(14):e34188. doi: 10.1016/j.heliyon.2024.e34188 (PMC11305241; doi:10.1016/j.heliyon.2024.e34188)
Supplement: Multimedia component 1 [file mmc1.pdf]

# Distributions

## Sex

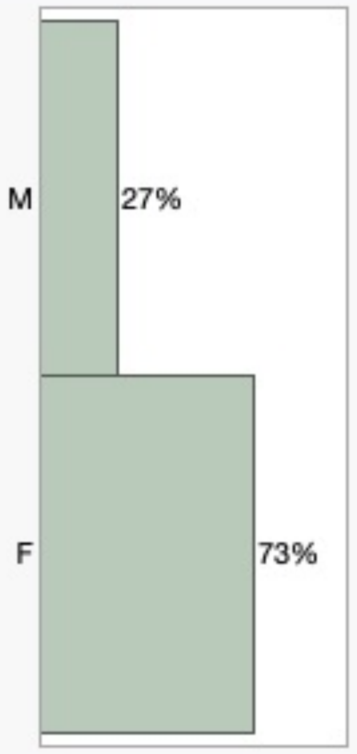


### Frequencies

| Level | Count | Prob |
|---|---|---|
| F | 44 | 0.73333 |
| M | 16 | 0.26667 |
| Total | 60 | 1.00000 |

N Missing 0

2 Levels

## Age

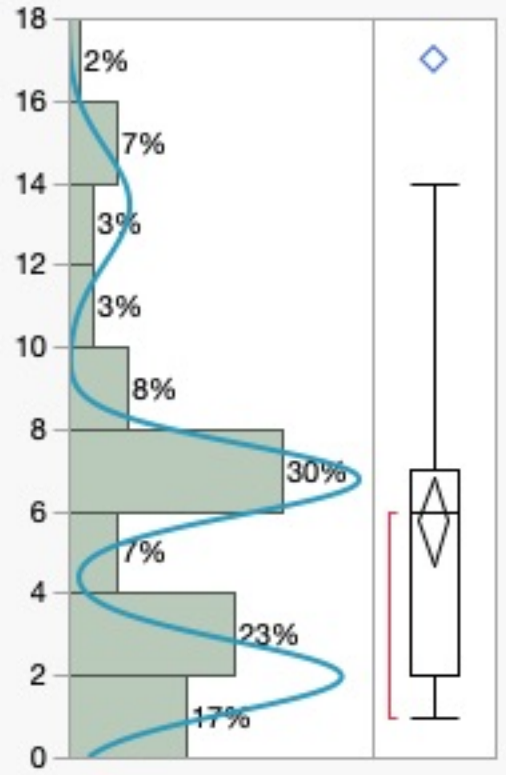


### Compare Distributions

| Show | Distribution | AICc | AICc Weight | BIC | -2*LogLikelihood |
|---|---|---|---|---|---|
| ☑ | Normal 3 Mixture | 310.67412 | 0.9963 | 324.60535 | 291.85059 |
| ☐ | Weibull | 323.99215 | 0.0013 | 327.97031 | 319.78162 |
| ☐ | SHASH | 324.05898 | 0.0012 | 331.70909 | 315.33171 |
| ☐ | Gamma | 324.40612 | 0.001 | 328.38428 | 320.19559 |
| ☐ | Lognormal | 328.76486 | 0.0001 | 332.74302 | 324.55433 |
| ☐ | Johnson Sb | 330.75349 | 4.3e-5 | 338.4036 | 322.02622 |
| ☐ | Exponential | 332.32027 | 2e-5 | 334.34565 | 330.25131 |
| ☐ | Normal 2 Mixture | 336.79508 | 2.1e-6 | 346.1557 | 325.68397 |
| ☐ | Normal | 342.49489 | 0 | 346.47306 | 338.28437 |
| ☐ | Student's t | 344.70452 | 0 | 350.55898 | 338.27595 |
| ☐ | Cauchy | 364.16309 | 0 | 368.14125 | 359.95256 |

### Quantiles

| | | |
|---|---|---|
| 100.0% | maximum | 17 |
| 99.5% | | 17 |
| 97.5% | | 15.425 |
| 90.0% | | 13 |
| 75.0% | quartile | 7 |
| 50.0% | median | 6 |
| 25.0% | quartile | 2 |
| 10.0% | | 1 |
| 2.5% | | 1 |
| 0.5% | | 1 |
| 0.0% | minimum | 1 |

### Summary Statistics

| | |
|---|---|
| Mean | 5.7666667 |
| Std Dev | 4.0895347 |
| Std Err Mean | 0.5279567 |
| Upper 95% Mean | 6.8231055 |
| Lower 95% Mean | 4.7102278 |
| N | 60 |
| N Missing | 0 |

### Fitted Normal 3 Mixture Distribution

| Parameter | | Estimate | Lower 95% | Upper 95% |
|---|---|---|---|---|
| Location | μ1 | 1.9613275 | 1.6227963 | 2.2998586 |
| Location | μ2 | 6.7639535 | 6.4391272 | 7.0887799 |
| Location | μ3 | 13.435105 | 12.531827 | 14.338384 |
| Dispersion | σ1 | 0.8631442 | 0.6035057 | 1.2344835 |
| Dispersion | σ2 | 0.8454875 | 0.5951777 | 1.2010684 |
| Dispersion | σ3 | 1.3826911 | 0.8484527 | 2.253319 |
| Probability | π1 | 0.4162118 | 0.2669456 | 0.5826075 |
| Probability | π2 | 0.4337673 | 0.2798923 | 0.6015678 |
| Probability | π3 | 0.150021 | 0.0756471 | 0.2757068 |

| Measures | |
|---|---|
| -2*LogLikelihood | 291.85059 |
| AICc | 310.67412 |
| BIC | 324.60535 |

## BCS

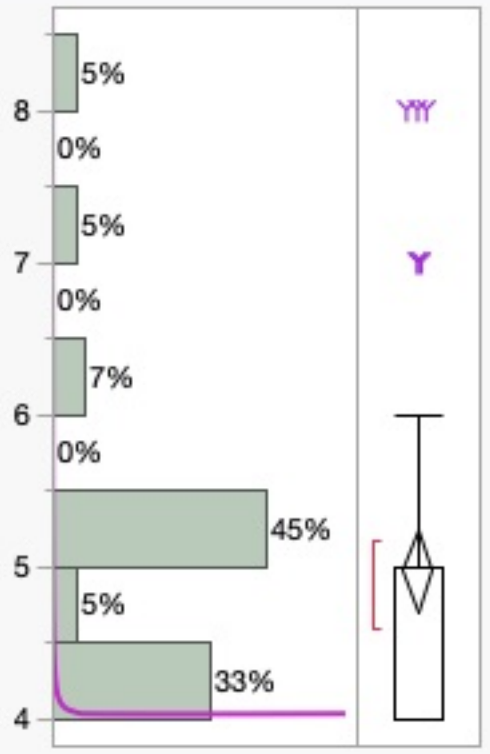


### Compare Distributions

| Show | Distribution | AICc | AICc Weight | BIC | -2*LogLikelihood |
|---|---|---|---|---|---|
| ☑ | SHASH | -6578.381 | 1 | -6570.731 | -6587.108 |
| ☐ | Johnson Sb | -274.5523 | 0 | -266.9022 | -283.2795 |
| ☐ | Normal 3 Mixture | 155.9765 | 0 | 169.90773 | 137.15297 |
| ☐ | Normal 2 Mixture | 157.24063 | 0 | 166.60124 | 146.12952 |
| ☐ | Lognormal | 163.8441 | 0 | 167.82226 | 159.63357 |
| ☐ | Gamma | 167.77981 | 0 | 171.75797 | 163.56928 |
| ☐ | Student's t | 168.58647 | 0 | 174.44094 | 162.1579 |
| ☐ | Cauchy | 169.12578 | 0 | 173.10394 | 164.91525 |
| ☐ | Normal | 177.60076 | 0 | 181.57892 | 173.39023 |
| ☐ | Weibull | 187.90272 | 0 | 191.88088 | 183.69219 |
| ☐ | Exponential | 314.60046 | 0 | 316.62584 | 312.5315 |

### Quantiles

| | | |
|---|---|---|
| 100.0% | maximum | 8 |
| 99.5% | | 8 |
| 97.5% | | 8 |
| 90.0% | | 6.9 |
| 75.0% | quartile | 5 |
| 50.0% | median | 5 |
| 25.0% | quartile | 4 |
| 10.0% | | 4 |
| 2.5% | | 4 |
| 0.5% | | 4 |
| 0.0% | minimum | 4 |

### Summary Statistics

| | |
|---|---|
| Mean | 4.9750187 |
| Std Dev | 1.0349089 |
| Std Err Mean | 0.1336062 |
| Upper 95% Mean | 5.242364 |
| Lower 95% Mean | 4.7076734 |
| N | 60 |
| N Missing | 0 |

### Fitted SHASH Distribution

| Parameter | | Estimate | Std Error | Lower 95% | Upper 95% |
|---|---|---|---|---|---|
| Shape | γ | -1.141447 | 0.0972559 | . | . |
| Shape | δ | 0.0115487 | 0.0007026 | . | . |
| Location | θ | 4 | 7.725e-78 | . | . |
| Scale | σ | 7.438e-80 | 0 | . | . |

| Measures | |
|---|---|
| -2*LogLikelihood | -6587.108 |
| AICc | -6578.381 |
| BIC | -6570.731 |

# Distributions

## Norm-GR

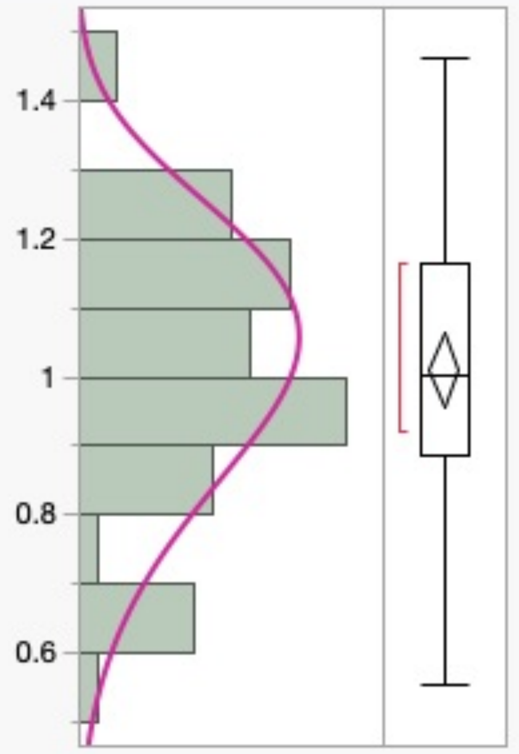


### Compare Distributions

| Show | Distribution | AICc ^ | AICc Weight | .2 .4 .6 .8 | BIC | -2*LogLikelihood |
|---|---|---|---|---|---|---|
| ☑ | Weibull | -17.81969 | 0.3597 | | -13.8789 | -22.03397 |
| | Normal 2 Mixture | -17.33853 | 0.2828 | | -8.08292 | -28.47061 |
| | Normal | -16.64709 | 0.2001 | | -12.7063 | -20.86138 |
| | Student's t | -14.43357 | 0.0662 | | -8.637321 | -20.86993 |
| | SHASH | -13.3466 | 0.0384 | | -5.777192 | -22.08734 |
| | Gamma | -13.31248 | 0.0378 | | -9.371687 | -17.52676 |
| | Lognormal | -10.49056 | 0.0092 | | -6.549775 | -14.70485 |
| | Normal 3 Mixture | -9.597723 | 0.0059 | | 4.1425763 | -28.47772 |
| | Johnson Sb | 3.5219003 | 8.3e-6 | | 11.091309 | -5.21884 |
| | Cauchy | 5.3611121 | 3.3e-6 | | 9.3019013 | 1.1468264 |
| | Exponential | 121.29257 | 0 | | 123.29993 | 119.22239 |

### Quantiles

| | | |
|---|---|---|
| 100.0% | maximum | 1.4596505 |
| 99.5% | | 1.4596505 |
| 97.5% | | 1.4379158 |
| 90.0% | | 1.2503401 |
| 75.0% | quartile | 1.1640669 |
| 50.0% | median | 1.0003743 |
| 25.0% | quartile | 0.8864969 |
| 10.0% | | 0.6618787 |
| 2.5% | | 0.5850737 |
| 0.5% | | 0.5556407 |
| 0.0% | minimum | 0.5556407 |

### Summary Statistics

| | |
|---|---|
| Mean | 1.0104131 |
| Std Dev | 0.2044861 |
| Std Err Mean | 0.0266218 |
| Upper 95% Mean | 1.0637025 |
| Lower 95% Mean | 0.9571237 |
| N | 59 |
| N Missing | 1 |

### Fitted Weibull Distribution

| Parameter | | Estimate | Std Error | Lower 95% | Upper 95% |
|---|---|---|---|---|---|
| Scale | α | 1.0919899 | 0.0260927 | 1.0402568 | 1.1442693 |
| Shape | β | 5.7410052 | 0.5807675 | 4.6578741 | 6.9326767 |

**Measures**

| | |
|---|---|
| -2*LogLikelihood | -22.03397 |
| AICc | -17.81969 |
| BIC | -13.8789 |

### Nonparametric One-Sided Tolerance Interval

| Proportion | Lower TI | Upper TI | 1-Alpha | Actual Confidence |
|---|---|---|---|---|
| 0.900 | 0.614507 | . | 0.950 | 0.9849 |

## Norm-TKT

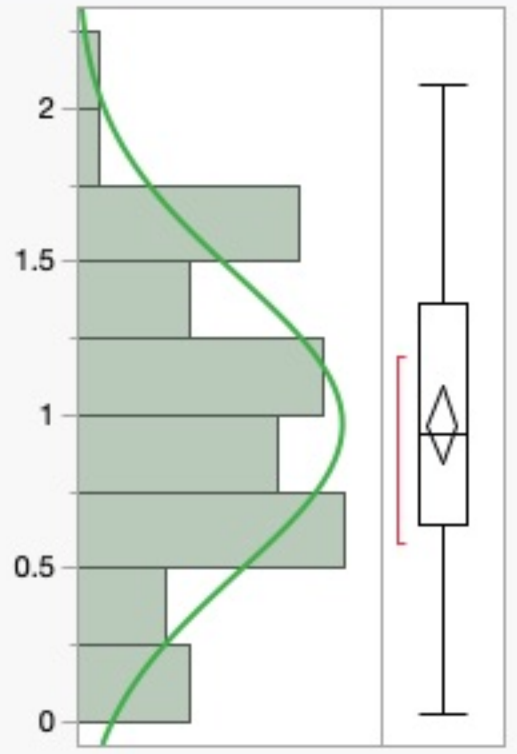


### Compare Distributions

| Show | Distribution | AICc ^ | AICc Weight | .2 .4 .6 .8 | BIC | -2*LogLikelihood |
|---|---|---|---|---|---|---|
| ☑ | Normal | 83.560133 | 0.4492 | | 87.462837 | 79.341951 |
| | Student's t | 85.777734 | 0.1482 | | 91.514619 | 79.33329 |
| | Weibull | 85.810092 | 0.1458 | | 89.712796 | 81.59191 |
| | SHASH | 86.228339 | 0.1183 | | 93.715394 | 77.473622 |
| | Normal 2 Mixture | 86.79089 | 0.0893 | | 95.939259 | 75.637044 |
| | Johnson Sb | 88.097436 | 0.0465 | | 95.584491 | 79.342719 |
| | Gamma | 94.206233 | 0.0022 | | 98.108937 | 89.988051 |
| | Normal 3 Mixture | 97.651657 | 0.0004 | | 111.19643 | 78.712882 |
| | Cauchy | 109.76573 | 0 | | 113.66843 | 105.54754 |
| | Exponential | 114.18465 | 0 | | 116.17366 | 112.11322 |
| | Lognormal | 114.7108 | 0 | | 118.61351 | 110.49262 |

### Quantiles

| | | |
|---|---|---|
| 100.0% | maximum | 2.0798548 |
| 99.5% | | 2.0798548 |
| 97.5% | | 1.9677457 |
| 90.0% | | 1.5911588 |
| 75.0% | quartile | 1.3607078 |
| 50.0% | median | 0.9393758 |
| 25.0% | quartile | 0.6441007 |
| 10.0% | | 0.266053 |
| 2.5% | | 0.0540684 |
| 0.5% | | 0.0299401 |
| 0.0% | minimum | 0.0299401 |

### Summary Statistics

| | |
|---|---|
| Mean | 0.9670484 |
| Std Dev | 0.4836782 |
| Std Err Mean | 0.0635101 |
| Upper 95% Mean | 1.0942251 |
| Lower 95% Mean | 0.8398717 |
| N | 58 |
| N Missing | 2 |

### Fitted Normal Distribution

| Parameter | | Estimate | Std Error | Lower 95% | Upper 95% |
|---|---|---|---|---|---|
| Location | μ | 0.9670484 | 0.0635101 | 0.8398717 | 1.0942251 |
| Dispersion | σ | 0.4836782 | 0.0455006 | 0.4089052 | 0.5921741 |

**Measures**

| | |
|---|---|
| -2*LogLikelihood | 79.341951 |
| AICc | 83.560133 |
| BIC | 87.462837 |

### One-Sided Tolerance Interval

| Proportion | Lower TI | Upper TI | 1-Alpha |
|---|---|---|---|
| 0.900 | 0.185719 | . | 0.950 |

## Norm-TKTwithTDP

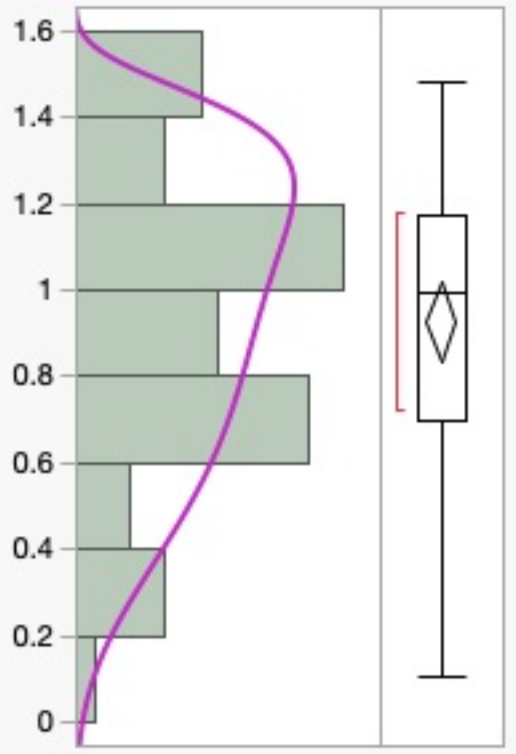


### Compare Distributions

| Show | Distribution | AICc ^ | AICc Weight | .2 .4 .6 .8 | BIC | -2*LogLikelihood |
|---|---|---|---|---|---|---|
| ☑ | SHASH | 44.450378 | 0.4257 | | 51.853352 | 35.681147 |
| | Johnson Sb | 46.011124 | 0.1951 | | 53.414098 | 37.241893 |
| | Normal | 46.22447 | 0.1753 | | 50.088351 | 42.002248 |
| | Weibull | 47.124377 | 0.1118 | | 50.988257 | 42.902155 |
| | Student's t | 48.44624 | 0.0577 | | 54.122564 | 41.99341 |
| | Normal 2 Mixture | 50.784989 | 0.0179 | | 59.823774 | 39.608518 |
| | Normal 3 Mixture | 51.003979 | 0.0161 | | 64.348389 | 32.003979 |
| | Gamma | 58.127705 | 0.0005 | | 61.991585 | 53.905483 |
| | Lognormal | 70.78988 | 0 | | 74.65376 | 66.567658 |
| | Cauchy | 71.814985 | 0 | | 75.678866 | 67.592763 |
| | Exponential | 107.58458 | 0 | | 109.5549 | 105.51185 |

### Quantiles

| | | |
|---|---|---|
| 100.0% | maximum | 1.4819555 |
| 99.5% | | 1.4819555 |
| 97.5% | | 1.4682614 |
| 90.0% | | 1.4397005 |
| 75.0% | quartile | 1.1734059 |
| 50.0% | median | 0.991383 |
| 25.0% | quartile | 0.6986143 |
| 10.0% | | 0.3553245 |
| 2.5% | | 0.1598781 |
| 0.5% | | 0.1080811 |
| 0.0% | minimum | 0.1080811 |

### Summary Statistics

| | |
|---|---|
| Mean | 0.928247 |
| Std Dev | 0.3528452 |
| Std Err Mean | 0.0467355 |
| Upper 95% Mean | 1.0218694 |
| Lower 95% Mean | 0.8346246 |
| N | 57 |
| N Missing | 3 |

### Fitted SHASH Distribution

| Parameter | | Estimate | Std Error | Lower 95% | Upper 95% |
|---|---|---|---|---|---|
| Shape | γ | 8.1359151 | 25.579719 | -42.41808 | 58.689907 |
| Shape | δ | 3.3097111 | 1.7725571 | -0.166222 | 6.7856442 |
| Location | θ | 2.3742046 | 0.8660543 | 0.6720052 | 105.14091 |
| Scale | σ | 0.2410044 | 1.7030706 | -3.125018 | 3.6070268 |

**Measures**

| | |
|---|---|
| -2*LogLikelihood | 35.681147 |
| AICc | 44.450378 |
| BIC | 51.853352 |

### Nonparametric One-Sided Tolerance Interval

| Proportion | Lower TI | Upper TI | 1-Alpha | Actual Confidence |
|---|---|---|---|---|
| 0.900 | . | 1.451524 | 0.950 | 0.9819 |

# Distributions

## PAR

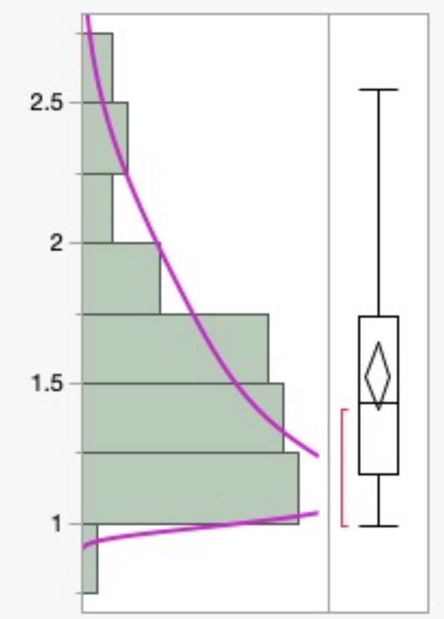


### Compare Distributions

| Show | Distribution | AICc ^ | AICc Weight | .2 .4 .6 .8 | BIC | -2*LogLikelihood |
|---|---|---|---|---|---|---|
| ☑ | SHASH | 49.207244 | 0.5836 | | 56.161155 | 40.35618 |
| ☐ | Lognormal | 51.538482 | 0.1819 | | 55.196072 | 47.293584 |
| ☐ | Johnson Sb | 53.126711 | 0.0822 | | 60.080622 | 44.275647 |
| ☐ | Gamma | 53.573624 | 0.0658 | | 57.231214 | 49.328726 |
| ☐ | Normal 3 Mixture | 54.274187 | 0.0463 | | 66.535299 | 34.92535 |
| ☐ | Normal 2 Mixture | 54.758507 | 0.0364 | | 63.210377 | 43.454159 |
| ☐ | Normal | 60.310229 | 0.0023 | | 63.967819 | 56.065331 |
| ☐ | Student's t | 62.479723 | 0.0008 | | 67.833454 | 55.979723 |
| ☐ | Weibull | 62.672258 | 0.0007 | | 66.329848 | 58.42736 |
| ☐ | Cauchy | 73.266938 | 3.5e-6 | | 76.924528 | 69.02204 |
| ☐ | Exponential | 150.05376 | 0 | | 151.925 | 147.97376 |

### Quantiles

| | | |
|---|---|---|
| 100.0% | maximum | 2.55 |
| 99.5% | | 2.55 |
| 97.5% | | 2.54025 |
| 90.0% | | 2.254 |
| 75.0% | quartile | 1.7360895 |
| 50.0% | median | 1.4303246 |
| 25.0% | quartile | 1.1775 |
| 10.0% | | 1.033 |
| 2.5% | | 0.99325 |
| 0.5% | | 0.99 |
| 0.0% | minimum | 0.99 |

### Summary Statistics

| | |
|---|---|
| Mean | 1.5262666 |
| Std Dev | 0.4188538 |
| Std Err Mean | 0.0580846 |
| Upper 95% Mean | 1.6428762 |
| Lower 95% Mean | 1.4096569 |
| N | 52 |
| N Missing | 8 |

### Fitted SHASH Distribution

| Parameter | | Estimate | Std Error | Lower 95% | Upper 95% |
|---|---|---|---|---|---|
| Shape | γ | -5.981666 | 17.694927 | -40.30904 | 28.345704 |
| Shape | δ | 1.4221626 | 0.3800561 | 0.6772625 | 2.8054328 |
| Location | θ | 0.7600198 | 0.1462608 | 0.129446 | 1.1655497 |
| Scale | σ | 0.0191323 | 0.2378308 | -0.441315 | 0.6130704 |

| Measures | |
|---|---|
| -2*LogLikelihood | 40.35618 |
| AICc | 49.207244 |
| BIC | 56.161155 |

### Nonparametric One-Sided Tolerance Interval

| Proportion | Lower TI | Upper TI | 1-Alpha | Actual Confidence |
|---|---|---|---|---|
| 0.900 | . | 2.52 | 0.950 | 0.9717 |

### Tolerance Intervals

| Proportion | Lower TI | Upper TI | 1-Alpha |
|---|---|---|---|
| 0.900 | 0.692702 | 2.359831 | 0.950 |

## Latency

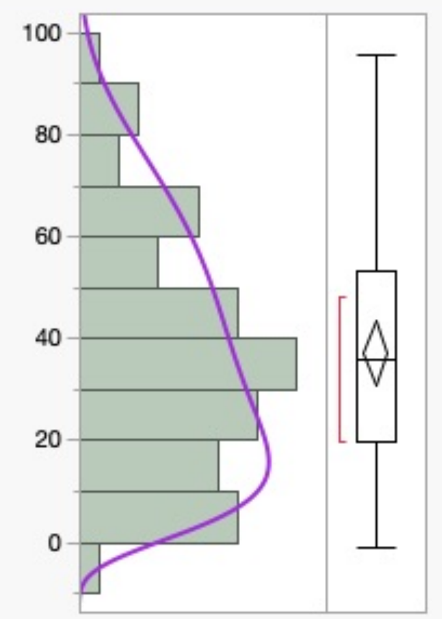


### Compare Distributions

| Show | Distribution | AICc ^ | AICc Weight | .2 .4 .6 .8 | BIC | -2*LogLikelihood |
|---|---|---|---|---|---|---|
| ☑ | SHASH | 553.70503 | 0.5422 | | 561.35513 | 544.97775 |
| ☐ | Normal | 554.92868 | 0.2941 | | 558.90684 | 550.71815 |
| ☐ | Student's t | 557.14006 | 0.0973 | | 562.99452 | 550.71148 |
| ☐ | Normal 2 Mixture | 559.41751 | 0.0312 | | 568.77813 | 548.3064 |
| ☐ | Normal 3 Mixture | 559.67609 | 0.0274 | | 573.60731 | 540.85256 |
| ☐ | Johnson Su | 562.16671 | 0.0079 | | 569.81682 | 553.43944 |
| ☐ | Cauchy | 576.56919 | 5.9e-6 | | 580.54736 | 572.35867 |

### Quantiles

| | | |
|---|---|---|
| 100.0% | maximum | 95.85 |
| 99.5% | | 95.85 |
| 97.5% | | 91.86525 |
| 90.0% | | 73.387 |
| 75.0% | quartile | 53.3875 |
| 50.0% | median | 35.82 |
| 25.0% | quartile | 19.7 |
| 10.0% | | 4.033 |
| 2.5% | | -0.42975 |
| 0.5% | | -0.96 |
| 0.0% | minimum | -0.96 |

### Summary Statistics

| | |
|---|---|
| Mean | 37.146583 |
| Std Dev | 24.015819 |
| Std Err Mean | 3.1004289 |
| Upper 95% Mean | 43.350526 |
| Lower 95% Mean | 30.942639 |
| N | 60 |
| N Missing | 0 |

### Fitted SHASH Distribution

| Parameter | | Estimate | Std Error | Lower 95% | Upper 95% |
|---|---|---|---|---|---|
| Shape | γ | -5.843516 | 14.714693 | -34.68378 | 22.996752 |
| Shape | δ | 2.9276872 | 1.4788861 | 0.882054 | 5.8262508 |
| Location | θ | -49.18306 | 60.540726 | -167.8407 | 69.474583 |
| Scale | σ | 22.91257 | 93.84398 | -161.0183 | 320.97165 |

| Measures | |
|---|---|
| -2*LogLikelihood | 544.97775 |
| AICc | 553.70503 |
| BIC | 561.35513 |

### Nonparametric One-Sided Tolerance Interval

| Proportion | Lower TI | Upper TI | 1-Alpha | Actual Confidence |
|---|---|---|---|---|
| 0.900 | . | 88.26 | 0.950 | 0.9862 |

## FAR

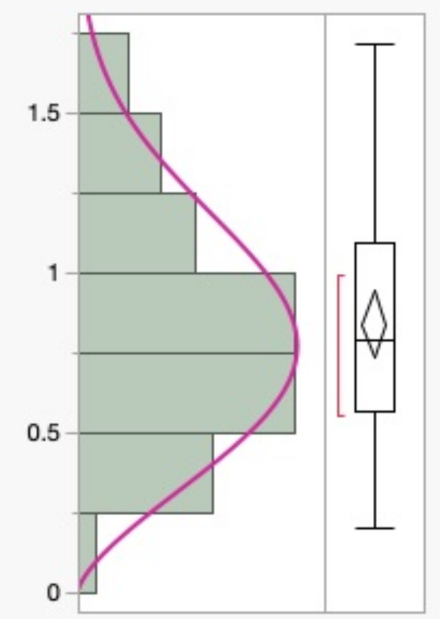


### Compare Distributions

| Show | Distribution | AICc ^ | AICc Weight | .2 .4 .6 .8 | BIC | -2*LogLikelihood |
|---|---|---|---|---|---|---|
| ☑ | Weibull | 41.872393 | 0.3837 | | 45.44112 | 37.617074 |
| ☐ | Gamma | 41.947108 | 0.3696 | | 45.515835 | 37.691789 |
| ☐ | Lognormal | 44.929864 | 0.0832 | | 48.498591 | 40.674545 |
| ☐ | Normal | 45.31402 | 0.0687 | | 48.882747 | 41.058701 |
| ☐ | SHASH | 45.815508 | 0.0534 | | 52.574711 | 36.926619 |
| ☐ | Student's t | 47.570327 | 0.0222 | | 52.784656 | 41.048587 |
| ☐ | Johnson Sb | 48.245673 | 0.0159 | | 55.004876 | 39.356784 |
| ☐ | Normal 2 Mixture | 51.413502 | 0.0033 | | 59.60998 | 40.049865 |
| ☐ | Normal 3 Mixture | 59.775156 | 5e-5 | | 71.559145 | 40.262961 |
| ☐ | Cauchy | 65.120813 | 3.4e-6 | | 68.68954 | 60.865494 |
| ☐ | Exponential | 84.602427 | 0 | | 86.431116 | 82.519093 |

### Quantiles

| | | |
|---|---|---|
| 100.0% | maximum | 1.7155409 |
| 99.5% | | 1.7155409 |
| 97.5% | | 1.7081086 |
| 90.0% | | 1.3292458 |
| 75.0% | quartile | 1.0955295 |
| 50.0% | median | 0.7877242 |
| 25.0% | quartile | 0.5672096 |
| 10.0% | | 0.3701009 |
| 2.5% | | 0.2225336 |
| 0.5% | | 0.2006057 |
| 0.0% | minimum | 0.2006057 |

### Summary Statistics

| | |
|---|---|
| Mean | 0.8396173 |
| Std Dev | 0.3684864 |
| Std Err Mean | 0.0521118 |
| Upper 95% Mean | 0.94434 |
| Lower 95% Mean | 0.7348946 |
| N | 50 |
| N Missing | 10 |

### Fitted Weibull Distribution

| Parameter | | Estimate | Std Error | Lower 95% | Upper 95% |
|---|---|---|---|---|---|
| Scale | α | 0.9484936 | 0.0572489 | 0.83858 | 1.0674997 |
| Shape | β | 2.4737444 | 0.2704347 | 1.9720056 | 3.0317954 |

| Measures | |
|---|---|
| -2*LogLikelihood | 37.617074 |
| AICc | 41.872393 |
| BIC | 45.44112 |

### Nonparametric One-Sided Tolerance Interval

| Proportion | Lower TI | Upper TI | 1-Alpha | Actual Confidence |
|---|---|---|---|---|
| 0.900 | . | 1.688514 | 0.950 | 0.9662 |

## SAD

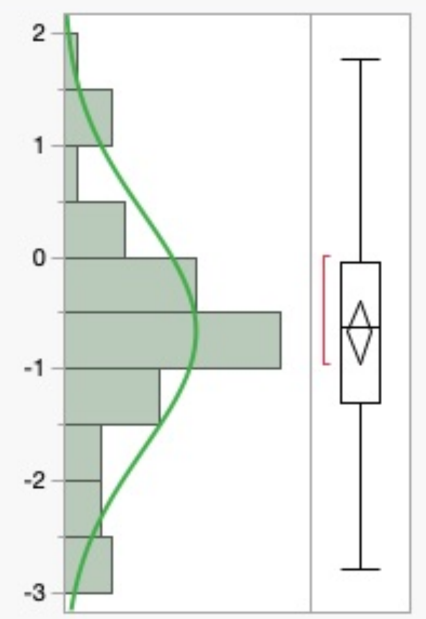


### Compare Distributions

| Show | Distribution | AICc ^ | AICc Weight | .2 .4 .6 .8 | BIC | -2*LogLikelihood |
|---|---|---|---|---|---|---|
| ☑ | Normal | 173.16562 | 0.477 | | 177.06832 | 168.94744 |
| ☐ | Student's t | 175.37156 | 0.1583 | | 181.10844 | 168.92712 |
| ☐ | Normal 2 Mixture | 175.57258 | 0.1432 | | 184.72095 | 164.41874 |
| ☐ | Normal 3 Mixture | 176.03873 | 0.1134 | | 189.5835 | 157.09995 |
| ☐ | SHASH | 176.3399 | 0.0975 | | 183.82695 | 167.58518 |
| ☐ | Johnson Su | 180.92094 | 0.0099 | | 188.408 | 172.16622 |
| ☐ | Cauchy | 186.05291 | 0.0008 | | 189.95561 | 181.83473 |

### Quantiles

| | | |
|---|---|---|
| 100.0% | maximum | 1.7660016 |
| 99.5% | | 1.7660016 |
| 97.5% | | 1.635413 |
| 90.0% | | 0.6841171 |
| 75.0% | quartile | -0.04584 |
| 50.0% | median | -0.619128 |
| 25.0% | quartile | -1.296948 |
| 10.0% | | -2.196614 |
| 2.5% | | -2.78576 |
| 0.5% | | -2.78576 |
| 0.0% | minimum | -2.78576 |

### Summary Statistics

| | |
|---|---|
| Mean | -0.670574 |
| Std Dev | 1.0472059 |
| Std Err Mean | 0.1375049 |
| Upper 95% Mean | -0.395226 |
| Lower 95% Mean | -0.945923 |
| N | 58 |
| N Missing | 2 |

### Fitted Normal Distribution

| Parameter | | Estimate | Std Error | Lower 95% | Upper 95% |
|---|---|---|---|---|---|
| Location | μ | -0.670574 | 0.1375049 | -0.945923 | -0.395226 |
| Dispersion | σ | 1.0472059 | 0.0985128 | 0.8853158 | 1.282109 |

| Measures | |
|---|---|
| -2*LogLikelihood | 168.94744 |
| AICc | 173.16562 |
| BIC | 177.06832 |

### One-Sided Tolerance Interval

| Proportion | Lower TI | Upper TI | 1-Alpha |
|---|---|---|---|
| 0.900 | . | 1.021074 | 0.950 |

## SAR

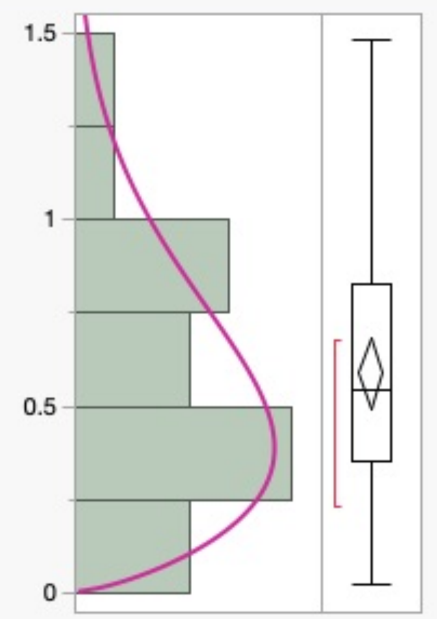


### Compare Distributions

| Show | Distribution | AICc ^ | AICc Weight | .2 .4 .6 .8 | BIC | -2*LogLikelihood |
|---|---|---|---|---|---|---|
| ☑ | Weibull | 36.262104 | 0.6839 | | 39.962688 | 32.022104 |
| ☐ | Gamma | 39.431259 | 0.1402 | | 43.131843 | 35.191259 |
| ☐ | SHASH | 40.888371 | 0.0677 | | 47.936205 | 32.055037 |
| ☐ | Johnson Sb | 41.060957 | 0.0621 | | 48.108791 | 32.227623 |
| ☐ | Normal | 42.550062 | 0.0295 | | 46.250646 | 38.310062 |
| ☐ | Student's t | 44.790321 | 0.0096 | | 50.2114 | 38.300525 |
| ☐ | Normal 2 Mixture | 45.638069 | 0.0063 | | 54.212933 | 34.361473 |
| ☐ | Exponential | 51.817465 | 0.0003 | | 53.709325 | 49.739033 |
| ☐ | Lognormal | 52.167118 | 0.0002 | | 55.867702 | 47.927118 |
| ☐ | Normal 3 Mixture | 52.835496 | 0.0002 | | 65.325104 | 33.562769 |
| ☐ | Cauchy | 64.954784 | 0 | | 68.655368 | 60.714784 |

### Quantiles

| | | |
|---|---|---|
| 100.0% | maximum | 1.48069 |
| 99.5% | | 1.48069 |
| 97.5% | | 1.4448804 |
| 90.0% | | 1.0443605 |
| 75.0% | quartile | 0.8273883 |
| 50.0% | median | 0.544529 |
| 25.0% | quartile | 0.3539003 |
| 10.0% | | 0.1297753 |
| 2.5% | | 0.0317853 |
| 0.5% | | 0.0242327 |
| 0.0% | minimum | 0.0242327 |

### Summary Statistics

| | |
|---|---|
| Mean | 0.5881555 |
| Std Dev | 0.3506072 |
| Std Err Mean | 0.0481596 |
| Upper 95% Mean | 0.6847948 |
| Lower 95% Mean | 0.4915162 |
| N | 53 |
| N Missing | 7 |

### Fitted Weibull Distribution

| Parameter | | Estimate | Std Error | Lower 95% | Upper 95% |
|---|---|---|---|---|---|
| Scale | α | 0.6561163 | 0.0559227 | 0.5513681 | 0.775666 |
| Shape | β | 1.6901834 | 0.1872928 | 1.3442956 | 2.0783407 |

| Measures | |
|---|---|
| -2*LogLikelihood | 32.022104 |
| AICc | 36.262104 |
| BIC | 39.962688 |

### Nonparametric One-Sided Tolerance Interval

| Proportion | Lower TI | Upper TI | 1-Alpha | Actual Confidence |
|---|---|---|---|---|
| 0.900 | . | 1.378377 | 0.950 | 0.9741 |

# Principal Components: on Correlations

Variance estimation: Row-wise

## Correlations

| | Age | BCS | GR | TKT | TKT with TDP | PAR | Latency | FAR | SAD | SAR |
|---|---|---|---|---|---|---|---|---|---|---|
| Age | 1.0000 | 0.2393 | -0.2713 | -0.3931 | -0.3920 | 0.0886 | 0.1353 | 0.1592 | 0.0815 | 0.0608 |
| BCS | 0.2393 | 1.0000 | -0.0278 | -0.0056 | -0.0779 | 0.0253 | 0.0170 | -0.0191 | 0.0146 | -0.0187 |
| GR | -0.2713 | -0.0278 | 1.0000 | 0.3584 | 0.2336 | -0.1395 | -0.1611 | -0.0083 | 0.0574 | 0.0405 |
| TKT | -0.3931 | -0.0056 | 0.3584 | 1.0000 | 0.6930 | -0.4711 | -0.5414 | -0.3342 | 0.0421 | -0.0881 |
| TKT with TDP | -0.3920 | -0.0779 | 0.2336 | 0.6930 | 1.0000 | 0.2181 | 0.1212 | -0.5025 | -0.4743 | -0.4508 |
| PAR | 0.0886 | 0.0253 | -0.1395 | -0.4711 | 0.2181 | 1.0000 | 0.9154 | -0.1656 | -0.7036 | -0.4861 |
| Latency | 0.1353 | 0.0170 | -0.1611 | -0.5414 | 0.1212 | 0.9154 | 1.0000 | -0.0247 | -0.5329 | -0.4012 |
| FAR | 0.1592 | -0.0191 | -0.0083 | -0.3342 | -0.5025 | -0.1656 | -0.0247 | 1.0000 | 0.8024 | 0.9037 |
| SAD | 0.0815 | 0.0146 | 0.0574 | 0.0421 | -0.4743 | -0.7036 | -0.5329 | 0.8024 | 1.0000 | 0.9302 |
| SAR | 0.0608 | -0.0187 | 0.0405 | -0.0881 | -0.4508 | -0.4861 | -0.4012 | 0.9037 | 0.9302 | 1.0000 |

The correlations are estimated by Row-wise method.

## Summary Plots

| Number | Eigenvalue |
|---|---|
| 1 | 3.672390 |
| 2 | 2.679999 |
| 3 | 1.222962 |
| 4 | 0.977984 |
| 5 | 0.685982 |
| 6 | 0.578124 |
| 7 | 0.113233 |
| 8 | 0.053256 |
| 9 | 0.012935 |
| 10 | 0.003136 |

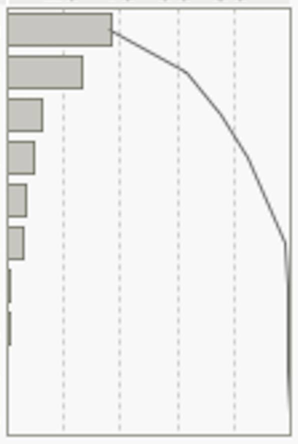


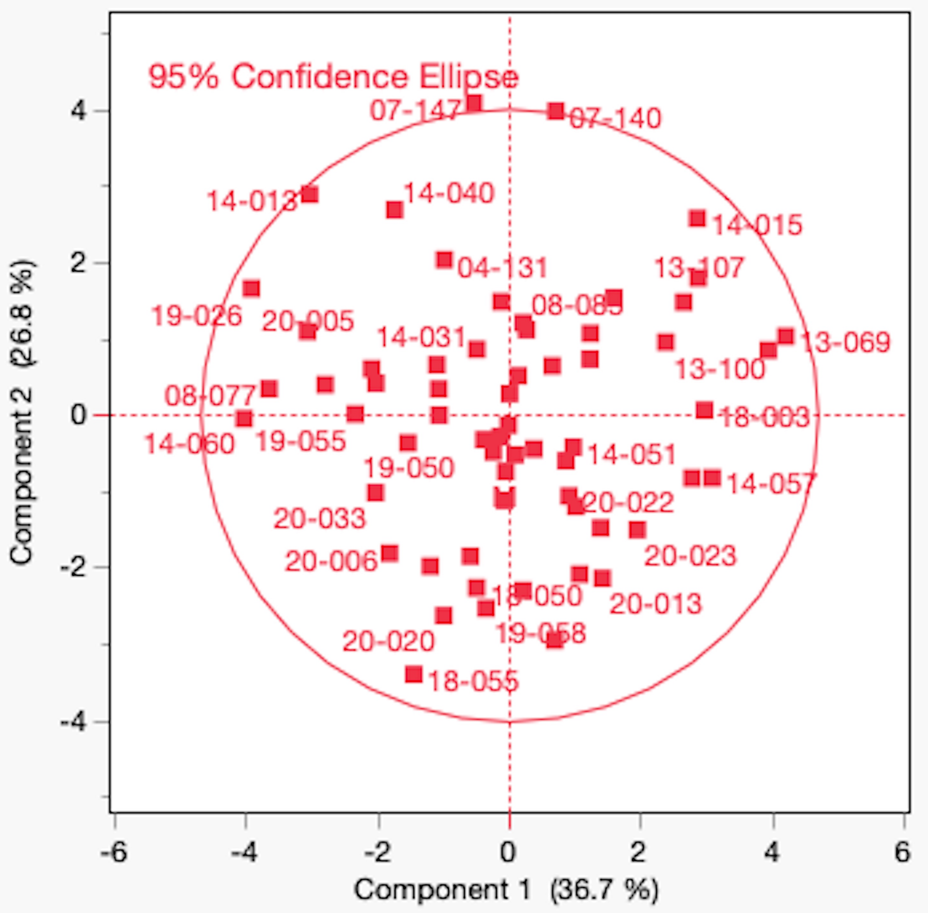


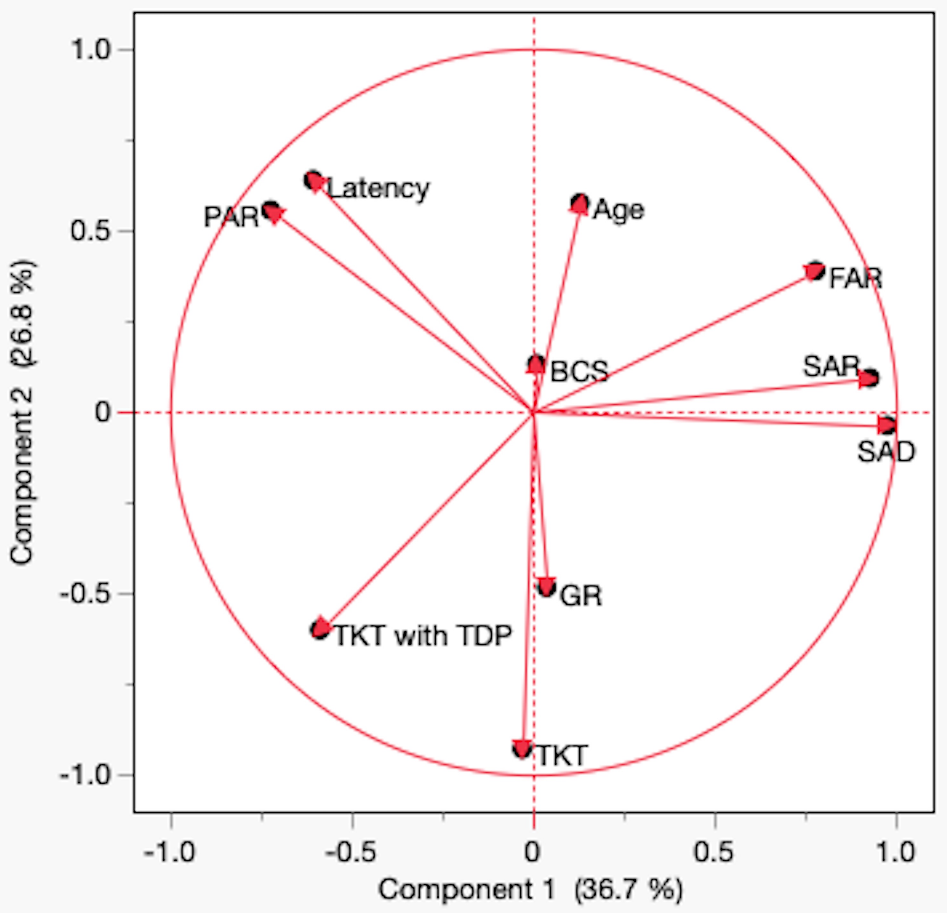


Label variables

Select component Component 1 Component 2

## Partial Contribution of Variables

| | Prin1 | Prin2 | Prin3 | Prin4 | Prin5 | Prin6 | Prin7 | Prin8 | Prin9 | Prin10 |
|---|---|---|---|---|---|---|---|---|---|---|
| Age | 0.46086 | 12.26917 | 21.86301 | 0.25512 | 8.09420 | 56.85459 | 0.14582 | 0.01582 | 0.00557 | 0.03583 |
| BCS | 0.00329 | 0.62800 | 33.47776 | 46.83482 | 6.81413 | 11.88579 | 0.01408 | 0.18602 | 0.13894 | 0.01718 |
| GR | 0.04002 | 8.77553 | 8.91768 | 31.52899 | 50.38687 | 0.02058 | 0.00448 | 0.31525 | 0.00677 | 0.00382 |
| TKT | 0.02273 | 32.25703 | 0.91696 | 1.01835 | 5.31430 | 8.89421 | 1.70345 | 43.15397 | 5.17450 | 1.54448 |
| TKT with TDP | 9.36051 | 13.52586 | 2.24752 | 3.38538 | 16.35277 | 17.88352 | 0.79244 | 29.97437 | 5.39066 | 1.08698 |
| PAR | 14.18575 | 11.45186 | 7.04518 | 4.44559 | 1.31762 | 0.37807 | 21.59338 | 9.74354 | 7.11880 | 22.72022 |
| Latency | 9.96501 | 15.13852 | 9.27430 | 5.15112 | 1.79489 | 0.86441 | 40.71153 | 0.05694 | 5.73449 | 11.30879 |
| FAR | 16.52231 | 5.58199 | 11.54381 | 5.24048 | 4.26260 | 1.86741 | 0.02518 | 10.08608 | 44.57776 | 0.29239 |
| SAD | 25.95418 | 0.05823 | 0.35429 | 0.45300 | 1.45082 | 0.57657 | 17.50346 | 1.99201 | 7.67185 | 43.98558 |
| SAR | 23.48534 | 0.31381 | 4.35949 | 1.68714 | 4.21179 | 0.77485 | 17.50619 | 4.47600 | 24.18066 | 19.00474 |

## Plot of Partial Contribution of Variables

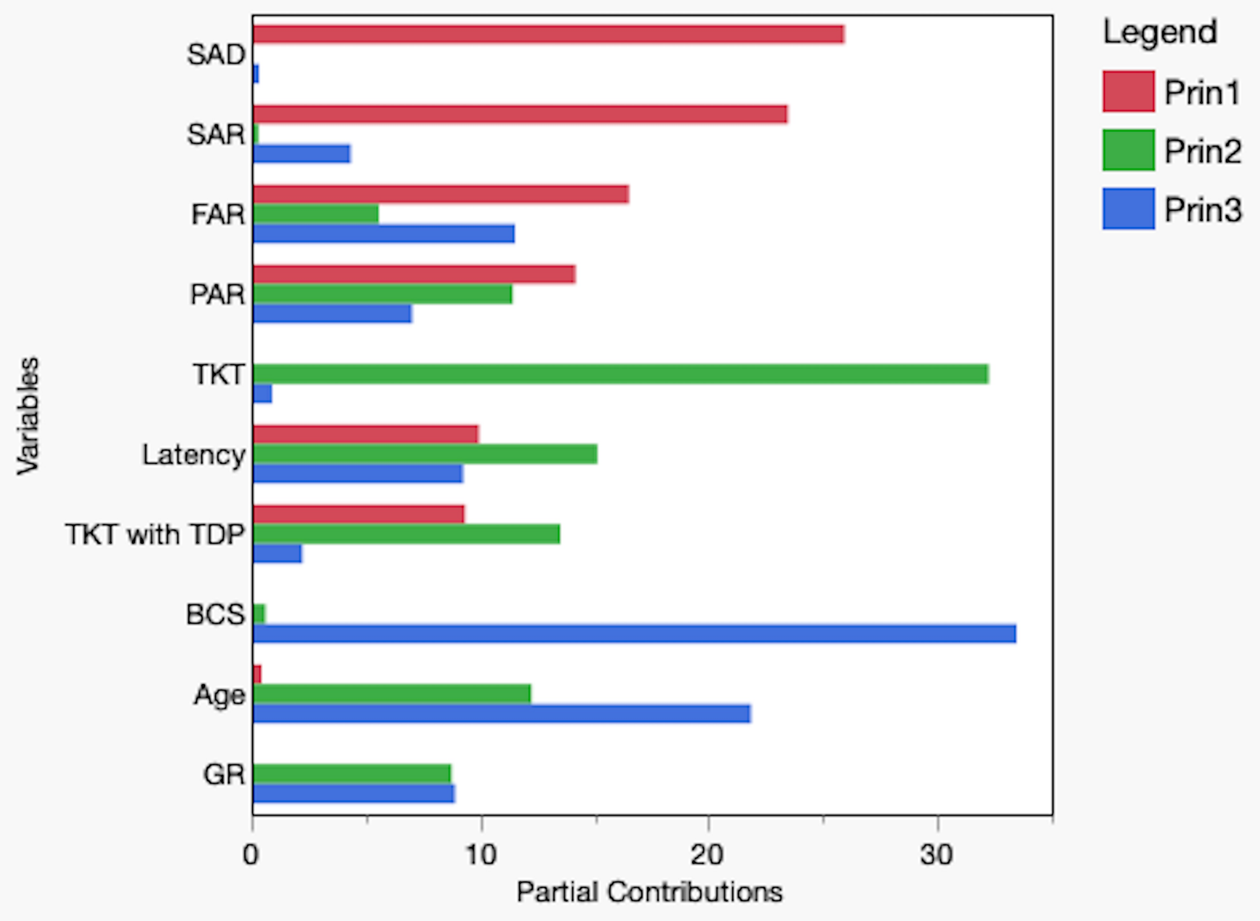


Number of Components 3
